# Mechano-metabolic axis involves mitochondrial responses via BMP/Smad regulatory effect on cyclophilin D

**DOI:** 10.1038/s41413-026-00580-y

**Published:** 2026-09-02

**Authors:** Rubens Sautchuk, Josaranie Nieves Santana, Chen Yu, Hani Awad, Roman A. Eliseev

**Affiliations:** 1 Center for Musculoskeletal Research, University of Rochester, Rochester, NY USA; 2 Department of Biomedical Engineering, University of Rochester, Rochester, NY USA; 3 Department of Pharmacology & Physiology, University of Rochester, Rochester, NY USA; 4 Department of Pathology, University of Rochester, Rochester, NY USA

**Keywords:** Bone, Energy metabolism

## Abstract

Mechanical stimulation is fundamental for anabolism in various tissues, including bone. Importantly, mechanical stimulation has been shown to induce reprogramming of cell metabolism, likely to adjust it to increased anabolism. The signal transduction within this mechano-metabolic axis is incompletely understood. Aiming to delineate such signal transduction, we exposed osteoprogenitors to fluid shear stress (FSS) and assessed cell signaling and bioenergetics. The BMP/Smad pathway is known to be activated by mechanical stress, and we indeed detected its activation by FSS. We previously reported that BMP downregulates cyclophilin D (CypD), an opener of the mitochondrial permeability transition pore (MPTP). Downregulation of CypD/MPTP improves mitochondrial inner membrane integrity and therefore oxidative function. We found that in FSS-stimulated cells, CypD was indeed downregulated and mitochondria were activated in a BMP-dependent manner. Meanwhile, the osteogenic effect of FSS was dependent on CypD downregulation and mitochondrial responses. To confirm our in vitro results in vivo, we optimized a novel model of tooth extraction-mediated unloading of craniofacial bones in mice. Such unloading led to bone loss and upregulation of CypD in the affected bone. Osteoblast-specific deletion of CypD protected against unloading-mediated bone loss, while CypD re-expression in these mice restored bone loss. In sum, we here present new evidence that the mechano-metabolic axis in osteogenic cells involves BMP/Smad-mediated downregulation of CypD and CypD-dependent mitochondrial responses. Such regulation is important for the osteoanabolic effect of mechanical stimulation. Our data also suggest that targeting CypD can be an effective strategy to prevent bone loss caused by unloading due to immobility, space flight, or tooth extraction.

## Introduction

Various tissues, including musculoskeletal, cardiovascular, and others, experience mechanical stimuli, such as fluid shear stress (FSS), compression, and stretching. Mechanical signals provide important cues for mechanosensitive cells in these tissues, stimulating their proliferation, differentiation, anabolism, and other functions.^1^ Cell energy metabolism and, in particular, the mitochondrial compartment, is influenced by mechanical stimulation.^2^ This phenomenon is termed “mechano-metabolism” and is thought to play a key role in the adaptation of mechanosensitive cells to changes in physical parameters of their microenvironment.^1,2^ However, there is no clear understanding of how mechanical cues are transduced to mitochondria. Activation of Piezo1 and 2 mechanoreceptors triggers cytosolic Ca^2+^ transients that may lead to mitochondrial Ca^2+^ uptake needed for mitochondrial TCA cycle activity.^3,4^ However, long-term mitochondrial responses may involve mechanisms other than short-term Ca^2+^ spikes. Our goal was to discover such mechanisms.

As a model, we used osteoprogenitors in vitro and craniofacial bones in vivo. Mechanical stress maintains bone remodeling balance by stimulating anabolic osteoblasts (OB) and inhibiting catabolic osteoclasts.^5–7^ Craniofacial and alveolar bones are known for their plasticity and strong response to mechanical stimuli.^8^ We have previously shown such plasticity in human subjects.^7,9^ Mechanical loading provides cues for activation and synchronization of hundreds of sites of bone remodeling and growth during postnatal development.^7,8^ Conversely, unloading inhibits osteogenic BMP/Smad signaling and OB differentiation, increases bone resorption,^5^ and is considered as a causal factor for the onset of osteoporosis.^6^ One of the most common bone disuse models is hindlimb suspension, a challenging and highly stressful procedure for animals. Tooth extraction is an alternative model as it depletes mechanical stimuli on alveolar bone and results in loss of bone volume and mineral content, as was observed in several prior works.^10,11^

In our pursuit of connecting mechanical stimulation to mitochondria, we considered the following: (1) Mechanical stimulation activates mitochondrial bioenergetics. Activation of mitochondria promotes OB anabolism as shown by us and others;^12,13^ (2) Osteoinduction in response to mechanical stimuli is at least partially driven by activation of BMP/Smad pathway;^14,15^ (3) We have previously shown that BMP/Smad signaling transcriptionally represses *Ppif* gene encoding cyclophilin D (CypD),^16^ the only genetically proven positive regulator of the mitochondrial permeability transition pore (MPTP). MPTP is a large non-selective channel and a negative regulator of oxidative metabolism.^17^ In fact, OxPhos is activated while MPTP is inhibited via *Ppif* downregulation during OB differentiation.^16,18–20^ Such an inhibition has also been reported during neuronal and cardiomyocyte differentiation;^17,21^ (4) CypD knockout mice present with less osteoporosis burden and improved fracture healing.^16,22,23^ In contrast, mitochondrial dysfunction due to CypD/MPTP gain-of-function (GOF) results in impaired osteogenic commitment and OB function;^16,24–27^ (5) RNAseq data from our previous work showed that CypD-null osteoprogenitors present pro-osteogenic and anti-inflammatory gene signatures^23^ similar to cells subjected to mechanical stress.^5,28^ Based on the above, we hypothesized a crosstalk between mechanical stimuli and mitochondrial activation through CypD/MPTP regulation in osteogenic cells under the control of BMP/Smad signaling.

## Results

**Fig. 1:**
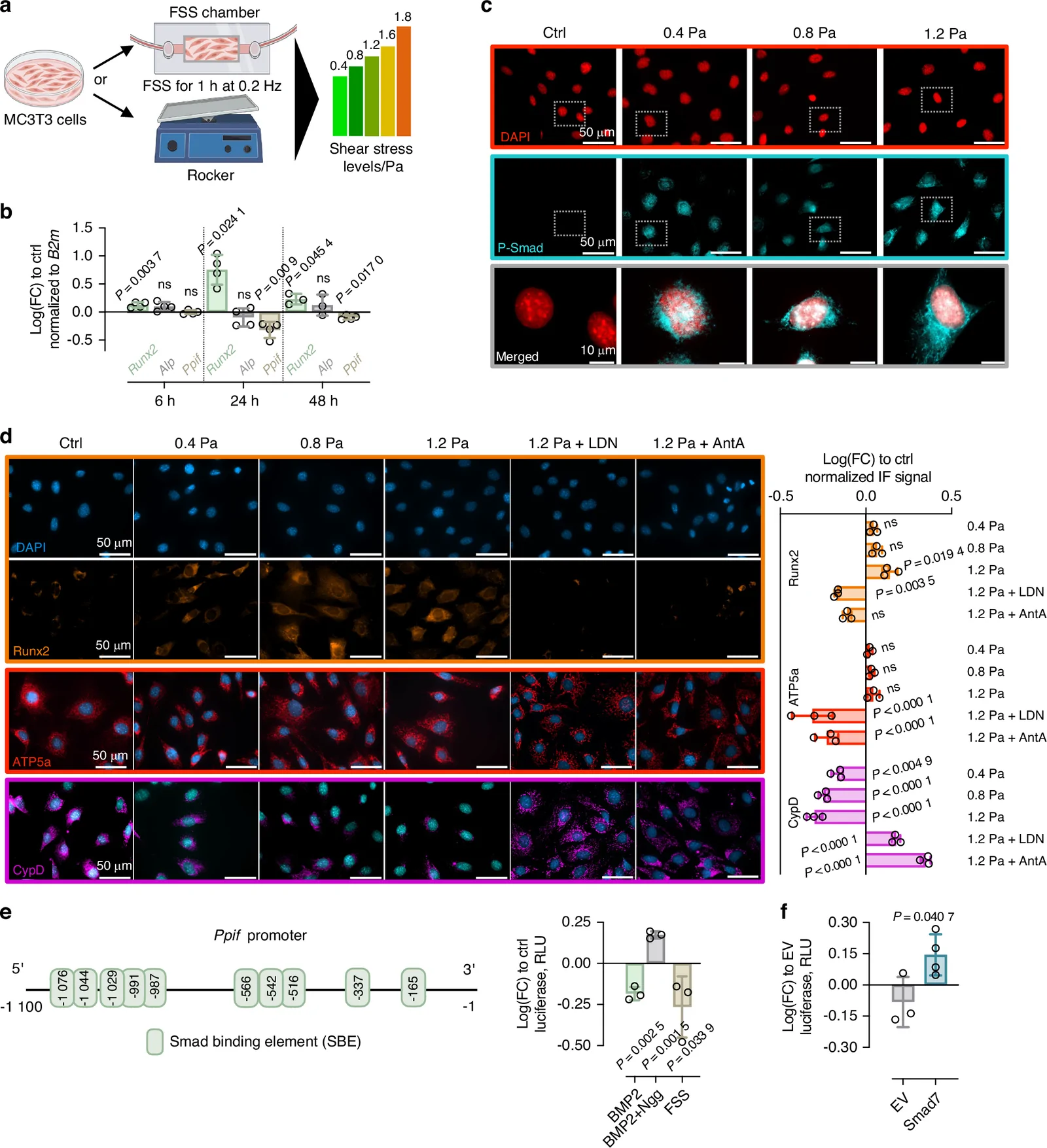
Flow shear stress activates the osteogenic cascade while downregulating the *Ppif* gene activity and mRNA expression. **a** Experimental design: MC3T3-E1 calvaria-derived cells were subjected to different intensities of mechanical stimulation ranging from 0.4 to 1.8 Pa of FSS intermittently at 0.2 Hz for 1 h. Non-induced cells were used as controls; **b** Real-time RT-qPCR data: RNA was collected at 6, 24, or 48 h after FSS induction. Twenty-four-hour time-point post-induction showed the highest levels of genetic response for *Runx2* and *Ppif* genes; **c** phospho-Smad (pSmad) IF-stained cells confirming BMP-Smad canonical activation (nuclear localization) upon FSS induction; **d** IF-stained panel of representative images comparing the protein levels of Runx2, ATP5a, and CypD at increasing FSS levels. No changes in ATP5a were found, whereas a linear decrease in CypD expression was observed. Both BMP receptor inhibitor, LDN, and the mitochondrial inhibitor, Antimycin A, perturbed the mitochondrial energy commitment, the initiation of osteogenic commitment, and reversed the levels of CypD expression; **e** dual luciferase reporter assay: diagram depicting the 1.1 kb *Ppif* promoter region cloned into the pGL4.10 vector. Cells were transfected with 1.1 Kb *Ppif*-Luc reporter vector and treated in BMP2-supplemented media with or without Noggin or FSS-induced. BMP2 or FSS induction decreased *Ppif* promoter activity, while Noggin rescued it; **f** cells transfected with the 1.1 kb *Ppif*-Luc reporter were co-transfected with *Smad7* or EV, and then FSS was induced. The inhibitory *Smad7* rescued the 1.1 kb *Ppif*-Luc reporter signal. Plots display the actual data points from independent biological replicates; Bar graphs report mean ± SD; *P* values determined by Tukey’s multiple comparison test following ordinary one-way ANOVA in multiple comparisons, or an unpaired *t*-test for a single continuous variable comparison

To test it, we first used cranial bone-derived osteoprogenitors, MC3T3, exposed to FSS since in vivo FSS generated by interstitial fluid flow is a major mechanical cue for various bone cells.^5,29^ FSS at different levels was generated using a parallel chamber or a moving rocker (Fig. 1a). Initially, we applied 1.2 Pa of FSS for 1 h at 0.2 Hz to mimic the cyclic stimulation observed in vivo. Cells were harvested at 6, 24, and 48 h post-FSS. We found that *Ppif* mRNA was, in fact, downregulated, whereas an osteogenic marker, *Runx2*, a readout of Smad activity, upregulated (Fig. 1b). Since gene expression results showed most pronounced changes at 24 h time-point, we subsequently harvested cells 24 h post-FSS. We observed that *Smad1* is upregulated along with *Runx2*, further proof of BMP/Smad activity (Fig. S1a). No significant changes were detected in cell viability or proliferation (Fig. S1b). To confirm BMP/Smad pathway activation post-FSS, we performed phospho-Smad immunofluorescence staining and observed its nuclear localization at different FSS levels (Fig. 1c). This indicates that our stress-induction model is sufficient to trigger a downstream Smad-dependent osteogenic cascade. Consistently, osteogenic markers were induced post FSS, such as Alkaline Phosphatase (ALP) staining and Runx2 immunofluorescence, peaking at 1.2 Pa (Figs. S1c, d and 1d) and corroborating previous publications showing a stress threshold for optimal mechano-induced osteoinduction.^29^ Within this optimal FSS intensity range, CypD protein expression progressively decreased (Fig. 1d), while no changes in ATP5a levels (a marker of mitochondrial content) were observed.

To determine whether the effect of FSS on CypD is BMP-dependent, we exposed cells to FSS at 1.2 Pa in the presence of BMP receptor inhibitor, LDN193189 (LDN). Runx2 was significantly downregulated in the presence of LDN (Fig. 1d), confirming inhibition of BMP signaling even in the presence of mechanical stimulation. We further observed that LDN not just canceled but completely reversed the inhibitory effect of FSS on CypD (Fig. 1d). This observation confirms the involvement of BMP signaling in the inhibitory effect of FSS on CypD. Interestingly, LDN also downregulated ATP5a and, therefore, mitochondrial content. We also wanted to find out if mitochondrial activity is required for the stimulatory effect of FSS on BMP/Smad signaling and exposed cells to FSS at 1.2 Pa in the presence of the mitochondrial Complex III inhibitor, Antimycin A (AntA). AntA reversed the stimulatory effect of FSS on Runx2, indicating its dependence on mitochondrial activity. AntA also decreased mitochondrial content (ATP5a), which is consistent with previous reports showing downregulation of expression of mitochondrial respiratory chain proteins by AntA.^30^ Upregulation of CypD by AntA was likely due to inhibition of BMP/Smad signaling shown by downregulation of Runx2 after such treatment (Fig. 1d). In sum, these experiments show that mechanical stimulation of osteogenic cells by FSS leads to activation of BMP/Smad signaling and BMP-dependent downregulation of CypD expression.

To evaluate the effect of unloading on *Ppif*, we analyzed microgravity data from NASA (https://genelab-data.ndc.nasa.gov/genelab/accession/GLDS-29/) and found that osteoprogenitors or OBs under microgravity, a proxy of unloading, present with higher *Ppif* expression compared to cells exposed to fluid pressure mimicking earth gravity or cells on earth (Fig. S1e). Not surprisingly, microgravity is shown to cause mitochondrial dysfunction in humans and mice, presenting with a metabolomic signature consistent with premature aging and oxidative stress.^31^ Similarly, we previously reported that CypD is upregulated in aged bones^16^ and shows a causal role in metabolic dysfunction, oxidative stress, and degeneration in bone.^32^ Therefore, we sought to investigate whether CypD GOF would disrupt osteoinduction post FSS. We used the pCMV6-*caPpif* vector that expresses the K166Q constitutively active CypD mutant or the empty vector (EV) to transfect MC3T3 cells. In GOF cells compared to EV-controls, *Ppif* expression was maintained even after FSS, disrupting *Smad1* and *Runx2* upregulation and, thus, the osteogenic cascade (Fig. S1f). We previously reported the presence of multiple Smad-binding elements (SBE) in the *Ppif* gene promoter and characterized the BMP/Smad-dependent pathway as a *Ppif* transcriptional repressor.^16^ Since post-FSS osteoinduction involved BMP/Smad signaling,^14,33^ we investigated whether FSS is sufficient to repress *Ppif*. We used a previously constructed by us *Ppif* promoter-reporter, *Ppif*-Luc (pGL4.10 luciferase backbone), in MC3T3 cells exposed to FSS. The specificity of our *Ppif*-Luc reporter was tested by co-transfecting it or its mutated version with no functional SBEs with pCMV6-*Smad1*. The Luc reporter with the native *Ppif* promoter showed a downregulated signal compared to the mutated one for pCMV6-*Smad1*-transfected cells (Fig. S1g). Next, cells were either treated with BMP2 (positive control) with or without the BMP inhibitor Noggin (negative control) or FSS-induced and assayed for luciferase signal. Both BMP2 and FSS downregulated *Ppif*-Luc activity, whereas Noggin rescued *Ppif*-Luc activity (Fig. 1e). Additionally, cells were co-transfected with the *Ppif*-Luc reporter and either pCMV6-*Smad7* or pCMV6-EV-control and then exposed to FSS. Inhibitory to BMP/Smad signaling, Smad7 restored the luciferase signal, suggesting a rescue of *Ppif* promoter activity (Fig. 1f). These data indicate a mechanism linking mechanical stress-induced osteogenic signaling with mitochondrial function via BMP/Smad-mediated regulation of *Ppif* transcription.

**Fig. 2:**
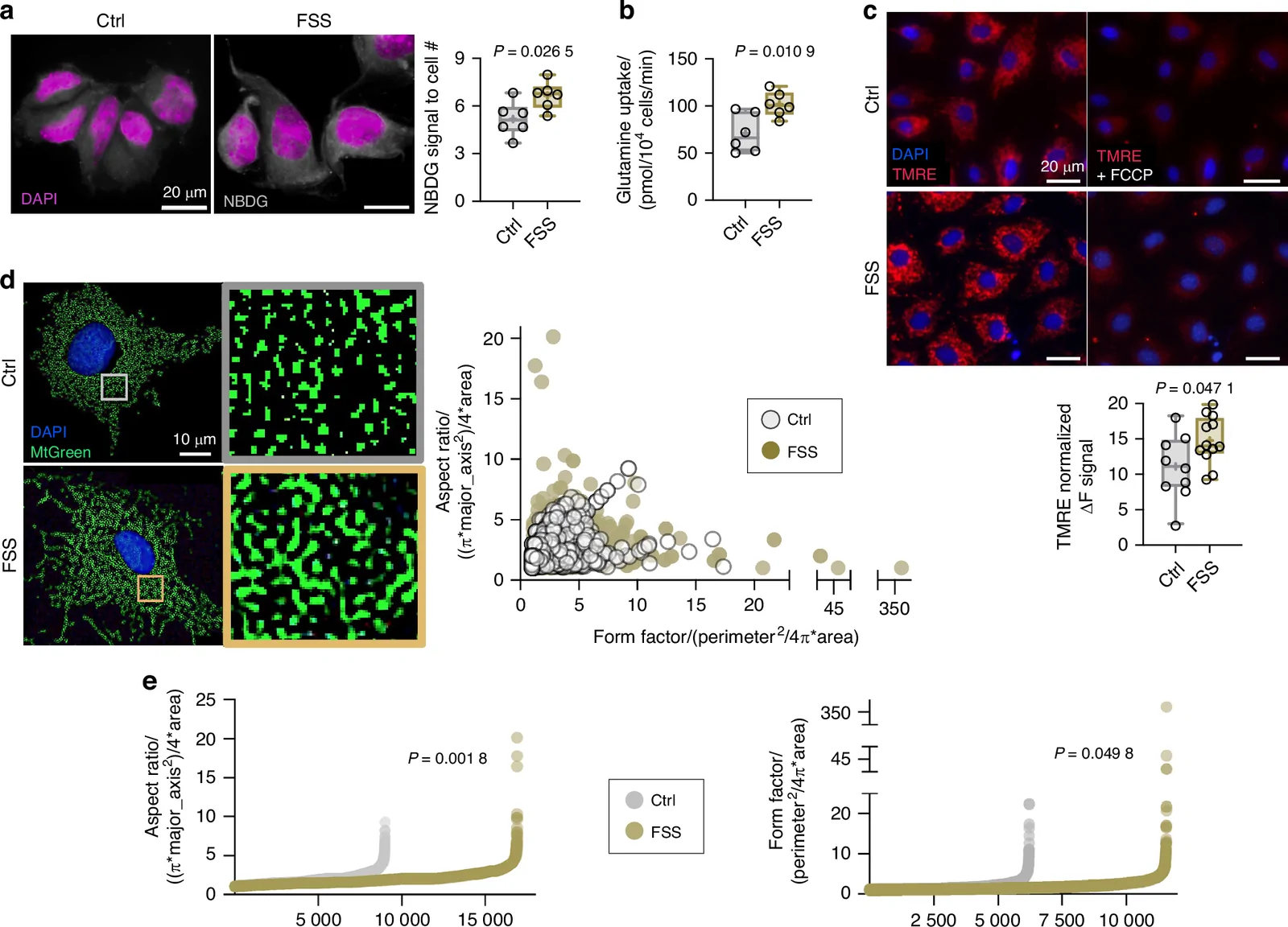
Flow shear stress increases mitochondrial OxPhos parameters. MC3T3-E1 calvaria-derived cells were subjected to 1.2 Pa of FSS intermittently at 0.2 Hz for 1 h and assayed 24 h later. Non-induced cells were used as controls. **a** FSS-induced cells showed higher glucose uptake, as revealed by NBDG signal intensity; **b** higher glutamine consumption; **c** representative images from control and FSS-induced cells stained with the nuclear stain, Hoechst33342, and the Δψm-dependent stain, TMRE. FCCP was used as a negative control for TMRE staining to derive the TMRE ΔF signal. Quantification shows increased TMRE ΔF signal, and therefore Δψm in FSS+ cells compared to controls; **d** representative images of FSS-exposed and control cells stained with MitoTracker Green. Mitochondria were identified in ImageJ using a convolution filter and thresholding for intensity and minimum pixel area. Particle analysis detected and measured individual mitochondria. The distribution of mitochondrial form and network complexity was plotted as the aspect ratio (relative elongation) over the form factor graph (network complexity). Each dot in the scatter plot represents an individual mitochondrion; a total of 16 913 mitochondria were analyzed in the control group and 11 564 in the FSS group. **e** Mitochondrial aspect ratio and form factor showed a significant increase in FSS cells compared to non-induced controls. Boxplots display the actual data points from independent biological replicates, showing median, mean, and range; *P* values determined by an unpaired *t*-test for a single continuous variable comparison

To further evaluate mechano-induced metabolic changes, we measured both glucose uptake using 2-NBDG and glutamine consumption post FSS vs. non-induced controls. We found increased glucose and glutamine uptake in mechano-induced cells (Fig. 2a, b). Since mechanical stress affects mitochondrial dynamics, we analyzed mitochondrial morphology in live cells using confocal microscopy. MC3T3 cells exposed to FSS were stained with the fluorescent nuclear stain, Hoechst33342, and various mitochondrial probes. First, we used a mitochondrial membrane potential (ΔΨm)-dependent stain, TMRE. FCCP, an uncoupler, was used as a negative control. FSS-induced cells showed a clear increase in TMRE signal and, therefore, Δψm and OxPhos capacity when compared to non-induced cells (Fig. 2c). Next, we calculated mitochondrial morphological parameters using MitoTracker Green staining (Fig. 2d). Although some absolute parameters did not change (Fig. S1h), FSS-induced cells showed mitochondrial elongation and higher network complexity compared to control cells (Fig. 2e). Taken together, our results suggest that mitochondria are activated upon mechanical loading and a profound bioenergetic response is triggered in osteogenic cells.

**Fig. 3:**
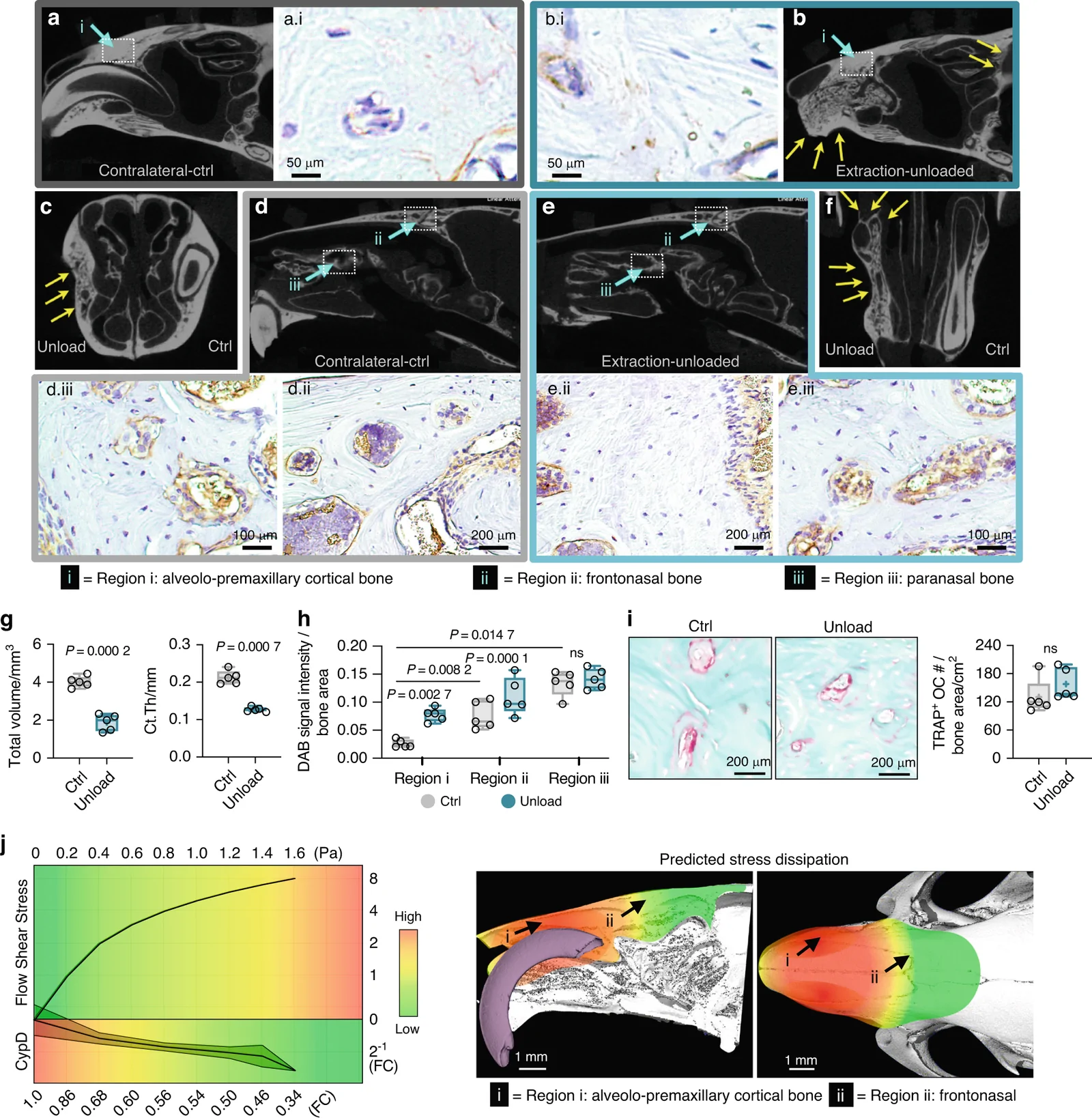
CypD expression is higher in the craniofacial bones after tooth extraction-mediated unloading. Unilateral maxillary incisor extraction was performed in 3-month-old C57BL/6J mice and followed up for 4 months. MicroCT data confirming the results presented in Fig. S2d–f, comparing the extraction-unloaded side to the contralateral-control and showing the spatial localization of representative IHC-stained slides probed for CypD. **a**, **b** MicroCT midsection view of maxillary incisor and extraction site, respectively; **c** MicroCT coronal view depicting maxillary, nasal, paranasal structures, and extraction-unloaded side vs. contralateral-control; **d**, **e** MicroCT sagittal view depicting paranasal bones and frontonasal suture from contralateral-control and extraction-unloaded side, respectively; **f** MicroCT axial view depicting alveolar, maxillary, and pre-maxilla bone regions. Yellow arrows point to extensive bone resorption areas. Blue arrows point to the representative spatial region of its respective histology sectioning; **g** total alveolar volume, and cortical thickness of alveolar bone are decreased in the extraction-unloaded side compared to contralateral-control; **h** alveolar cortical bone (a.i and b.i) and frontonasal region (d.ii and e.ii) propagate mechanical stimuli coming from incisor loading.^4^ Higher CypD signal is present in the tooth extraction-mediated unloading side in regions i and ii (b.i and e.ii) compared to the contralateral-control side (a.i and d.ii). Paranasal bones (d.iii and e.iii) receive no mechanical stimuli from incisor loading,^8^ no difference is found in CypD levels in the paranasal bone region (d.iii vs. e.iii); **i** TRAP staining representative images. Quantification comparing the extraction-unloaded to contralateral-control regions of alveolar cortical bone showed no differences in osteoclast activity; **j** intensity correlation map (data plotted from FSS over CypD protein IF-expression in vitro: Fig.1d and Fig. S1d) was used to build a 2D model. The intensity map was overlaid on midsagittal and axial microCT reconstruction images employing CypD downregulation fold change in vivo, and its spatial characterization (Fig. 3a, d, h) as anchors to derive a predictive 2D model for strain dissipation resulting from masticatory force-induced loading in the contralateral-control bone continuum. Boxplots display the actual data points from independent biological replicates, showing median, mean, and range; *P* values determined by Tukey’s multiple comparison test following ordinary one-way ANOVA in multiple comparisons, or an unpaired *t*-test for a single continuous variable comparison

To confirm the regulatory role of CypD in the connection of mechanical stress and mitochondrial function in vivo, we used a mouse model of tooth extraction, which is known to lead to unloading and bone loss in the craniofacial skeleton.^7–9^ Socket healing following tooth extraction takes ~28 days, during which minor loss of alveolar bone is observed, an event associated with post-traumatic inflammation and bone resorption.^11^ This short-term effect after incisor extraction has been well studied in mice.^34^ However, the long-term effect and continuous alveolar bone loss most likely associated with unloading following tooth extraction has not been formally described in such a model. We, therefore, performed unilateral maxillary incisor extraction in 3-month-old C57BL/6J mice (Fig. S2a, b) and followed it either for 28 days until complete socket healing (short-term) or 4 months (long-term). We first detected a short-term negative effect of tooth extraction on alveolar bone (Fig. S2c, d, white arrows and Fig. S2f, g), recapitulating bone loss during socket healing observed in previous studies.^33^ At 4 months post-extraction, there was extensive bone loss in the maxillary region (Fig. S2e, green arrows, and Fig. S2f, g). This finding strengthens our model’s applicability and reliability for studies of unloading-mediated loss of craniofacial bone. X-ray results were confirmed by micro-CT volumetric analysis comparing the extraction-unloaded side to the contralateral-control (Fig. 3a–c, f), pointing to a significant decrease in total volume and cortical thickness (Fig. 3g). Next, we performed immunohistochemistry (IHC) staining for CypD, having the unloaded and contralateral sides on the same slide. The regions expected to propagate mechanical loading coming from incisor stimulation showed increased CypD levels on the unloaded side (Figs. 3a.i, b.i, d.ii, e.ii, h and S3a–d). Conversely, regions described to receive stimulation from other means, such as paranasal bones,^8,35^ showed no differences in CypD levels between the unloaded and contralateral side (Figs. 3d.iii, e.iii, h and S3e, f), further confirming that CypD expression is controlled by mechanical cues in craniofacial structure. Interestingly, our data show that on the control non-extraction side, CypD level in bone tissue is directly correlated to the distance from the loading-site origin, and therefore inversely correlated to the predicted stress dissipated from incisor mechanical loading (Fig. S3g). This observation suggests that CypD levels are at least partially controlled by the loading force magnitude directly applied to or perceived by the tissue. To address this possibility, we plotted an intensity correlation map utilizing the in vitro data from Figs. 1d and S1e with additional data points for FSS level to CypD expression, which also showed an inverse correlation with mechanical stress (Fig. S3h). Using this 2D model, we applied the intensity model over the midsagittal and axial micro-CT 3D reconstruction, utilizing the CypD fold change in vivo data as anchors. This strategy allowed us to characterize the masticatory strain dissipation resulting from masticatory force-induced loading in the bone tissue (Fig. 3j). Interestingly, our strain predictive model, based on the levels of CypD expression, was very similar to that found in previous reports utilizing finite element analysis.^36,37^ Lastly, we found no differences in osteoclast activity when the extraction-unloaded side with higher CypD levels was compared to the contralateral-control showing a lower CypD signal (Figs. 3i and S3i). Our aggregated data directly relate the influence of mechanical stimuli on mitochondrial energy state in mechanosensitive tissue, i.e., bone, and position CypD as a potential therapeutic target to manipulate the effects of unloading.

**Fig. 4:**
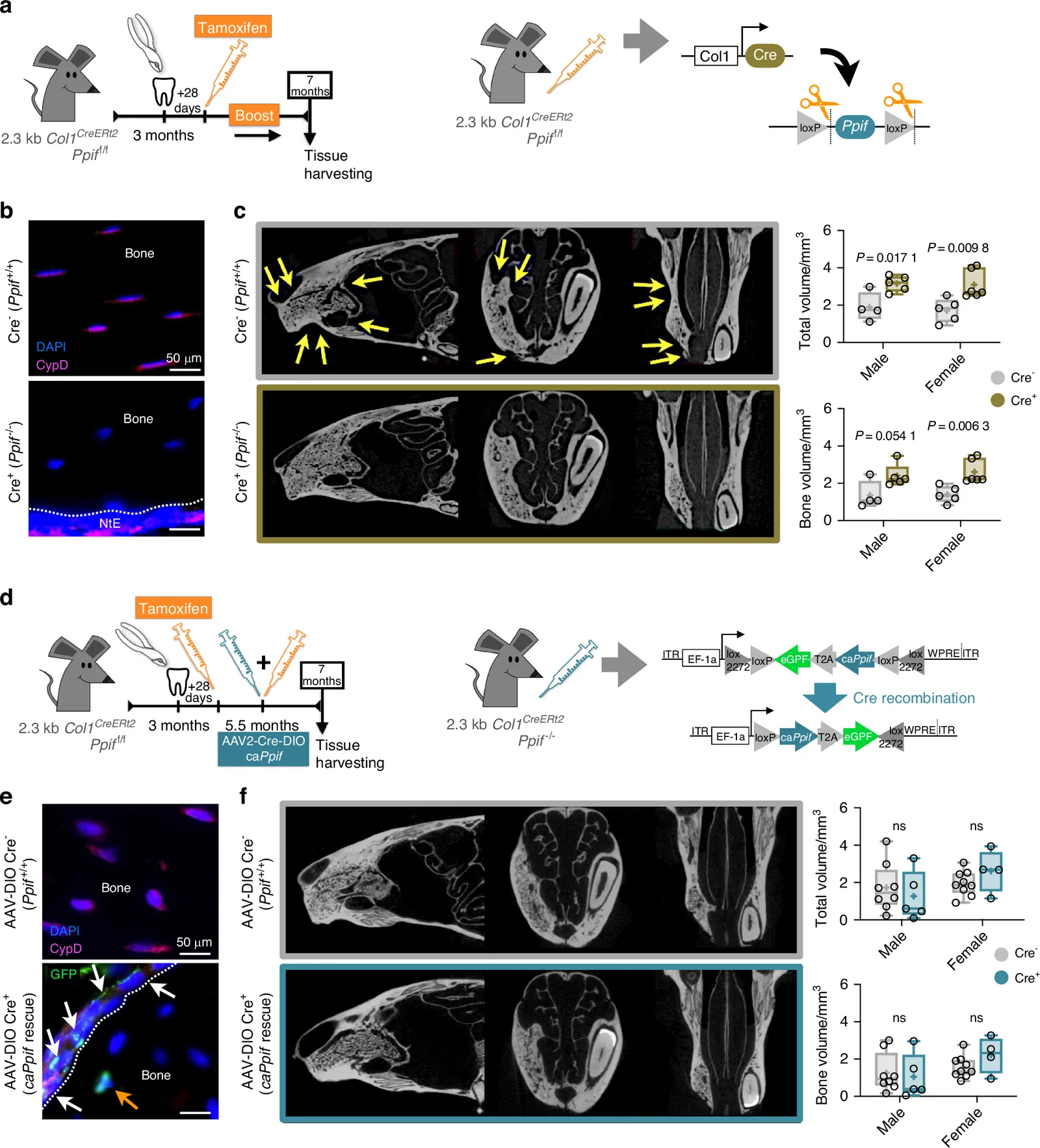
*Ppif* conditional knockout in osteoblasts is protective against disuse bone loss in craniofacial bones. **a** Experimental design and *Ppif* cKO strategy: Unilateral maxillary incisor extraction was performed in 3-month-old OB-specific inducible *Col1*^*CreERt2/+*^; *Ppif*^*f/f*^ mice. Recombination and, therefore, *Ppif* deletion was induced 28 days post-surgery, followed by a tamoxifen boost injection 1.5 months after initial recombination, and tissue harvested when mice reached 7 months of age; **b** CypD immunofluorescence signal is present in Cre^−^ bone samples, but it is absent only in the bone in Cre^+^ mice confirming the successful *Ppif* conditional deletion (NtE: nasoturbinate epithelium); **c** representative microCT images showed increased bone loss (depicted by the arrows) in alveolar, maxillary and pre-maxilla bones in Cre^−^ control mice compared to Cre^+^ *Ppif* cKO mice. Total alveolar volume and alveolar bone volume are decreased in control Cre^−^ mice compared to Cre^+^ *Ppif*^*−/−*^ mice; **d** experimental design, CypD rescue and recombination strategy: Unilateral maxillary incisor extraction was performed in 3-month-old OB-specific *Col1*^*CreERt2/+*^; *Ppif*^*f/f*^ mice. Cre-mediated recombination and thus, *Ppif* deletion was induced 28 days post-surgery. Virus intra-cortical alveolar injection was performed in the palatine bone of the tooth extraction side 45 days after the initial recombination. The gene of interest is inserted into the virus vector in antisense orientation and is flanked by double-floxed sites. In cells expressing Cre recombinase, the gene of interest is flipped and activated following recombination through tamoxifen injection; **e** eGFP signal confirming the successful viral infection and recombination in the maxillary alveolar bone of Cre^+^ mice 1.5 months after subperiosteal injection (white arrows: lining OBs, orange arrow: embedded osteocyte); **f** representative microCT images showing no differences in bone loss levels of alveolar, maxillary and pre-maxilla bones in Cre^−^ control mice compared to Cre^+^ mice after *caPpif* expression. Total alveolar volume and alveolar bone volume presented no differences in control AAV-DIO Cre^−^ mice compared to Cre^+^ CypD GOF. Boxplots display the actual data points from independent biological replicates, showing median, mean, and range; *P* values determined by an unpaired *t*-test for a single continuous variable comparison

We have shown before that global and OB-specific *Ppif* deletion protects against bone loss in aging.^16,22^ Additionally, RNAseq analysis from our previous work revealed that *Ppif* knock-out (KO) osteoprogenitors (bone marrow stromal cells, BMSC) present pro-osteogenic and anti-inflammatory gene signatures^23^ matching the genetic priming of osteogenic cells subjected to mechanical stress.^5,28^ In fact, we analyzed publicly available RNAseq data from a study that mechanically stimulated BMSCs^38^ and found a potential dynamic reciprocal interaction between mechanical stimulation and CypD expression (Fig. S4a, b). Therefore, CypD/MPTP regulation appears to be a part of mechanotransduction. Moreover, FSS decreases *Ppif* expression in vitro, and unloading increases CypD levels in vivo. We therefore tested if CypD loss-of-function can recapitulate the downstream pathway triggered by mechanical loading and offer protection against unloading-mediated bone loss. Unilateral maxillary incisor extraction was performed as described above on 3-month-old control (*Ppif*^*f/f*^) or OB-specific inducible *Ppif* deletion (2.3 Kb *Col1*^*CreERt2/+*^;*Ppif*^*f*/f^ or *Ppif* cKO) mice. Tamoxifen-induced recombination was performed after complete socket healing, at 28 days post-surgery, and again 45 days later. Sample collection was done at 4 months post-surgery (Fig. 4a). Successful *Ppif* cKO was confirmed by CypD IF staining. Cre^+^ mice showed CypD IF signal only in surrounding tissues (nasoturbinate epithelium) of nasomaxillary bone, whereas no IF signal was detected in the actual bone (Fig. 4b). MicroCT volumetric analysis was used to evaluate alveolar bone at the extraction site and surrounding maxillary bone. Extensive areas of bone loss were detected in control mice, whereas *Ppif* cKO mice showed little bone loss compared to their respective contralateral side (Fig. 4c). Accordingly, total alveolar region and alveolar bone volume were decreased in the tooth-extraction side in control mice compared to *Ppif* cKO mice, confirming that OB-specific *Ppif* deletion is protective against unloading-induced bone loss. No changes in other micro-CT parameters were observed between groups (Fig. S5a).

To further confirm our results, we investigated the effects of *Ppif* re-expression and GOF in the model described above. We used an AAV CRE-DIO^39^ viral delivery of a constitutively active K166Q CypD mutant (*caPpif*) along with the eGFP reporter previously developed by us.^16^ This system enables the expression of the gene of interest only in Cre^+^ cells, providing specificity (Fig. 4d). In our experimental design, OB-specific CypD deletion in 2.3 kb *Col1*^*CreERt2*^*;Ppif*^*f/f*^ mice was induced beginning at 28 days post-tooth extraction. Cre^−^ mice (*Ppif*^*f/f*^) were also injected with tamoxifen and used as controls. The AAV2-CRE-DIO-*caPpif*-eGFP vector was introduced via subperiosteal injection in palatal bone on the tooth-extraction side of the above mice 45 days after the initial recombination, and therefore, *Ppif* deletion. Forty-five days after the viral infection and recombination, tissue was harvested and analyzed (Fig. 4d). Positive eGFP signal was mostly found in bone-lining OBs in Cre^+^ mice, confirming *Ppif* conditional re-expression in vivo (Fig. 4e). Micro-CT analysis revealed that CypD re-expression and GOF rescued the increased levels of bone loss observed in Cre^−^ control mice (Fig. 4f). Despite the relatively high standard deviation found in the AAV-DIO cohort (Fig. S6), even when volume parameters were normalized to the contralateral side (Fig. S3j), it did not lead to statistical differences between Cre^−^ (*Ppif*^+/+^) and AAV-DIO Cre^−^ (*Ppif*^+/+^) mice (Fig. S3k). Additionally, this higher variability did not mask the differences in the bone response in *Ppif*-expressing tissue vs. *Ppif* deletion upon mechanical unloading (Fig. S3l). It therefore suggests that AAV-DIO injection and viral infection did not change the micro-CT parameters of the study at baseline. It also confirms that unloading-mediated bone loss is mediated by CypD expression and, consequently, MPTP-dependent mitochondrial metabolism in OBs. In sum, these data indicate the role of CypD/MPTP-dependent mitochondrial response in the degeneration of mechanosensitive tissue (bone) due to unloading.

## Discussion

Cell mechano-sensation cascade involves various interactors, such as the cytoskeleton, lipid bilayer, extracellular matrix, intracellular organelles, and others. How mechanical energy is transduced into metabolic responses and chemical energy in the form of ATP is not well characterized.^40^ Herein, we identified that FSS-induced mechanical stimulation downregulates CypD expression in osteoprogenitor cells while activating osteogenic marker gene expression and initiating the osteogenic cascade. Mechanical stimulation alone was sufficient to upregulate mitochondrial function and OxPhos parameters. We also successfully presented a tooth extraction-mediated unloading model to mimic disuse bone loss in craniofacial structure and found that unloading upregulates CypD expression in vivo and that OB-specific *Ppif* deletion can prevent bone loss in such a scenario. In general, we provided important details linking mechanical stimuli to mitochondrial responses.

In more detail, FSS-mediated downregulation of *Ppif* mRNA was accompanied by upregulation of osteogenic markers, *Smad1* and *Runx2*, target genes of the BMP/Smad pathway. Considering our prior report that BMP/Smad signaling transcriptionally represses *Ppif*,^16^ we hypothesized that this pathway is involved in the observed mechano-metabolic axis. In fact, mechanical loading has been shown before to promote osteogenic differentiation at least in part through BMP/Smad signaling.^14,15^ Accordingly, FSS downregulated not only *Ppif* mRNA and CypD protein levels but also *Ppif* promoter activity, in fact indicating transcriptional repression. Such a repression was reversed when FSS was accompanied by inhibition of BMP/Smad signaling, proving the key role of this pathway in the observed effects. Of note, mechanical stimulation was also shown to increase osteoblastogenesis through Wnt signaling;^41^ however, in our previous report, we demonstrated that Wnt signaling had no effect on *Ppif* regulation and expression.^16^ Additionally, FSS increased ΔΨm and promoted mitochondrial fusion and elongation, a readout of their activation and higher OxPhos levels.^18^ Downregulation of *Ppif*/CypD expression decreases MPTP activity and, as a consequence, increases the integrity of the inner mitochondrial membrane and coupling of the electron transport chain. This leads to higher OxPhos activity.^42^ These processes, in turn, require higher carbon flow into the Krebs cycle, ultimately boosting glucose uptake and glutamine anaplerosis as shown in FSS-induced cells. The observed activation of the osteogenic cascade upon BMP/Smad signaling also must sustain higher energy and biosynthetic demand as OB differentiation takes place.^31^ These mitochondrial and metabolic responses were crucial for FSS-mediated differentiation effects because concurrent inhibition of OxPhos reversed these effects. Therefore, we can conclude that mechanical stimulation activates mitochondrial function through BMP/Smad-mediated downregulation of *Ppif* transcription in order to support a higher OxPhos activity needed for OB differentiation. We also analyzed public data from the NASA space flight study and found that BMSCs under microgravity, a proxy of unloading, upregulate *Ppif* expression. In addition, microgravity was shown to cause mitochondrial dysfunction in humans and mice, presenting a metabolomic signature consistent with premature aging and oxidative stress,^31^ very similar to our findings in a previous work where we evaluated the effects of *Ppif* overexpression in osteogenic cells and bone tissue.^16^ Since craniofacial bones are very sensitive to changes in mechanical forces, we decided to use a model of continuous alveolar bone loss observed after dental extraction. This model affects cell populations that play a role in alveolar bone formation and homeostasis, while artificial means (such as osteointegrated titanium dental implants) capable of propagating mechanical forces within alveolar bone are sufficient to balance bone turnover. Therefore, we took advantage of a tooth extraction model to mimic the effects of disuse bone loss in craniofacial bones. We found higher CypD levels in the tooth extraction mediated-unloading side compared to the contralateral-control side. This result is in accordance with the data on BMSCs under microgravity, i.e., that *Ppif*/CypD expression has an inverse correlation with mechanical cues. Accordingly, we found that CypD level in bone tissue was directly correlated with the distance from the loading-site origin in the contralateral-control side in an exposure-response relationship. This finding strengthens the idea that mechanical stimulation is a very important modulator of bone metabolism and, therefore, bone morphological features and homeostasis. Thus, we can suggest that bone tissue, in general, responds to mechanical cues not only through signaling pathways such as Yap/Taz, Wnt, BMP/Smad, and others, but also through metabolic adaptations controlled by mitochondrial function and the *Ppif* regulatory axis.

Interestingly, transcriptomic data from our previous work showed that CypD KO BMSCs present pro-osteogenic and anti-inflammatory gene signatures matching the genetic priming of osteogenic cells subjected to mechanical stress. It suggests that *Ppif* downregulation recapitulates the downstream pathway triggered by mechanical loading and can potentially offer protection against bone loss. Using an OB-specific inducible *Ppif* deletion mouse model, we in fact demonstrated that *Ppif* ablation offers protection against unloading-induced bone loss after unilateral maxillary incisor extraction. Conversely, CypD GOF reversed the beneficial effects of its deletion and rescued the detrimental effects of unloading on bone. Although our findings are specific in terms of mouse model and type of bone being studied, our results can be potentially extrapolated to other models and tissues. For instance, activation of SIRT1/SIRT3 in mice significantly increases bone mass in the hind limb unloading model.^26^ CypD deacetylation is mediated by SIRT3, leading to a decrease in MPTP activity and consequently higher OxPhos.^43^ Therefore, it is possible to indirectly attribute the protective effect of SIRT1/SIRT3 activation against bone loss to CypD/MPTP downregulation as well.

Overall, our findings provide additional details in linking mechanical stimuli to mitochondrial function and how mechanosensitive tissue health can be affected by their interplay. CypD levels and expression can potentially be targeted to normalize homeostasis of tissues experiencing disuse. Specifically for craniofacial and alveolar bone, CypD can be the first therapeutic target against bone loss observed after dental extraction, and to prevent or mitigate craniofacial deformities by manipulating bone modeling and growth during postnatal development. Since the tooth extraction-mediated unloading model locally mimics disuse-mediated bone loss, similar to microgravity effects on bone, this study suggests a potential mechanism to control microgravity-induced bone loss. Mechanistically, our study proposes the following mechano-metabolic axis: mechanical stimulation → BMP/Smad signaling → downregulation of CypD → activation of mitochondria.

## Materials and methods

### Mouse strains

2.3 kb *Col1*^*CreERt2*^ mice were a kind gift from Dr. Ackert-Bicknell (formerly of the University of Rochester, originated in the Karsenty Lab at Columbia University Department of Genetics). *Ppif* floxed (*Ppif*^*f/f*^) mice were acquired from Jackson Laboratories (originated in the Korsmeyer lab at Dana Farber Cancer Institute, RRID: IMSR_JAX:005737) and bred in-house with 2.3 kb *Col1*^*CreERt2*^ mice to allow OB-specific CypD deletion. The C57BL/6J mouse strain was obtained from the Jackson Laboratory (RRID: IMSR_JAX:000664) and bred in-house. All mice were housed at 23 °C on a 12-h light/dark cycle with free access to water and PicoLab Rodent Diet 20 (LabDiet #5053, St. Louis, MO). Mice were in group housing when applicable, based on weaning. Testing naïve mice with an average weight of 28 g were used for experiments. The assessments of animal studies were performed in a blinded and coded manner.

### Mouse cell lines

Mouse calvarial bone-derived MC3T3-E1 cells were acquired from ATCC. Cells were expanded in sterile conditions and maintained in a 37 °C incubator at 5% CO_2_ in low glucose αMEM media (Gibco A10490–01) containing 1 mmol/L L-glutamine, ribonucleosides (0.01 g/L each), deoxyribonucleosides (0.01 g/L each), no ascorbic acid, 10% FBS (Gibco 10437–028) heat-inactivated for 30 min at 55 °C, and 1% Penn/Strep (Gibco 15140–122). Cells were maintained at passage numbers < 20 as recommended by the supplier and from previous handling experience. Cells have been authenticated by their osteogenic differentiation potential. They are mycoplasma-free. Experimental flow shear-stressed (FSS) cells were induced either in the FSS-chamber or on the rocker platform. Both methods showed P-Smad nuclear translocation, as shown in Fig. 1c. Real-time RT-qPCR and mitochondrial morphology and functional data were collected using an FSS-chamber, whereas protein IF data and glucose and glutamine assay results were acquired using the rocker platform to develop flow shear stress.

### Flow shear stress (FSS) chamber

MC3T3-E1 cells were cultured as described above. Fifty thousand cells/well were seeded onto a 2-well Nunc™ Lab-Tek™ II Chamber Slide™ and let grow for 48 h. For the experimental chamber slides, chambers were disassembled, and slides with cells were placed into the chambers designed to create a steady shear flow. Fluid flow was applied for 1 h to cells in a parallel plate flow chamber using a closed flow loop powered by a syringe pump (NE-1000 Multi-Phaser™, New Era Pump Systems Inc.). The cell monolayer was subject to 1.2 Pa of shear stress determined by the *Wall Shear Stress Equation*, through intermittent laminar flow at 0.2 Hz. The *Fluid Dynamics Continuity Equation for Isochoric Flow* was used to determine the flow rate in the syringe barrel to adjust the pump parameters. For control cells, non-induced chamber slides were disassembled, and slides with cells were placed into sham chambers, where no shear stress was produced.

#### Wall shear stress equation for a parallel plate channel

This equation was used to calculate the flow rate needed in the chamber to result 1.2 Pa of shear stress.

Chamber dimensions = 25 mm × 0.75 mm × 75 mm

$$\tau =\frac{6\mu Q}{b{h}^{2}}$$

*τ* = FSS (pressure)—Pa or N/m^2^ —> 1.2 Pa;

*μ* = media viscosity—Pa·s—> 0.001 Pa·s;

*b* = chamber base length—m—> 0.025 m;

*h* = chamber height length—m—> 0.000 75 m;

*Q* = flow rate (volume/time)—m^3^/s—> *Q* = 0.000 002 812 5 m^3^/s —> 168.75 mL/min.

#### Fluid dynamics continuity equation for isochoric (incompressible) flow

It was used to determine the flow rate in the syringe barrel to set up the syringe pump parameters.

$${{\boldsymbol{S}}}_{{\bf{1}}}{{\boldsymbol{Q}}}_{{\bf{1}}}={{\boldsymbol{S}}}_{{\bf{2}}}{{\boldsymbol{Q}}}_{{\bf{2}}}({\rm{area}}\;{\times}\,{\rm{flow}}\;{\rm{rate}})$$

60 mL Syringe radius = 13.295 mm (diameter = 26.59 mm);

60 mL Syringe barrel cross-sectional area = 555.298 mm^2^;

Chamber cross-sectional area = 18.75 mm^2^;

Chamber flow rate = 168.75 mL/min;

*Q*_1_ (60 mL syringe flow rate) = 5.697 mL/min.

### FSS developed on the rocker platform

MC3T3-E1 cells were cultured as described. Sixty thousand cells/well were seeded onto a 6-well plate to maintain the same cell density as in cells plated on chamber slides and let grow for 48 h. For the experimental plates, fluid flow was applied for 1 h to the cells by placing the plate on a rocking platform, with the plate’s long axis perpendicular to the direction of the rocker movement at 37 °C in 5% CO_2_ humid incubator. FSS is created by the gravitational motion of the media moving back and forth over the cell monolayer. The magnitude of FSS is calculated and can be adjusted using a lubrication-based model described by the formula:

$$\tau =\frac{{\uppi}\mu{\theta }_{\max }}{2{\delta }^{2}T}$$

*τ* = FSS (pressure);

*μ* = media viscosity;

*θ*_max_ = maximum flip angle;

*δ* = ratio of fluid depth to well length;

*T* = time for one cycle.

To develop FSS higher than 1.2 Pa, 8% polyvinylpyrrolidone (PVP) was added to the media to increase the media viscosity to 4.044 ± 0.011 mPa·s (approximately that of blood). Alkaline Phosphatase (AlkPhos) staining (Thermo NBT/BCIP 1-step #34042) was performed on NBF-fixed cell monolayers. Staining intensity was measured using ImageJ and normalized to cell count. Control and Control-PVP cells showed no differences for AlkPhos staining and IF-stained Runx2, ATP5a, and CypD levels.

### Real-time RT-qPCR

Total RNA was isolated using the RNeasy kit (Qiagen 74106) and reverse transcribed into cDNA using the qScript cDNA synthesis kit (Quanta 95048–500). cDNA was subjected to real-time RT-PCR. The primer pairs used for genes of interest are outlined in the Key Resources Table. Real-time RT-PCR was performed in the RotorGene system (Qiagen) using SYBR Green (Quanta 95072–012). *Smad1* and *Runx2* gene expression, normalized to *B2m*, was used to confirm the initiation of osteogenic differentiation.

### Cloning of the *Ppif* promoter into the luciferase dual reporter and reporter activity assay

Mouse *Ppif* promoter fragments containing the −1.1 to −0.1 kb region were PCR amplified from purified C3H/HeJ mouse DNA. Primers were designed to introduce CTCGAG XhoI 5′ and AAGCTT HindIII 3′ flanking sequences. For *Ppif* promoter mutagenesis, point mutations for each Smad Binding Element found in the 1.1 kb *Ppif* promoter region were designed to substitute either a cytosine or guanine for an adenosine nucleotide in the sense strand, and therefore a complementary thymine base in the antisense strand. CTCGAG XhoI 5′ and AAGCTT HindIII 3′ flanking sequences were also introduced in the 1.1 kb mutated *Ppif* promoter, and a synthetic sequence fragment was ordered from IDT (IDT, gBlocks Gene Fragments). The fragments were then subcloned into the XhoI 3’/HindIII 5’ site of the promoterless pGL4.10 luciferase reporter vector. The correct insert orientation of the resulting promoter reporters was verified by sequencing. To evaluate the *Ppif* promoter activity, the constructed *Ppif*-Luc reporter was transfected into MC3T3-E1 cells at 0.8 μg per well in 12-well plates. The promoterless Renilla luciferase vector pRL (Promega, Madison, WI) was co-transfected at 50 ng per well as a reference. Smad1 activity was further activated with either 0.8 μg/mL pCMV-*Smad1* co-transfection, or 50 ng/mL BMP2. BMP receptor activation was perturbed by the BMP inhibitor, Noggin (R&D Systems 1967-NG-025/CF) at 0.1 µg/mL. The role of FSS mechanotransduction in repressing *Ppif* transcription was delineated by co-transfecting inhibitory *Smad7* using 0.8 μg/mL pCMV-*Smad7* vector. Firefly and Renilla luciferase activities were measured using an Optocomp 1 luminometer and a Dual Luciferase Reporter Assay System (Promega) according to the manufacturer’s protocol. The firefly luciferase signal was normalized to the Renilla luciferase signal and expressed as relative luminescence units fold change to the pCMV empty vector control.

### Western blot

Cells were lysed in a lysis buffer containing protease inhibitors and subjected to 4%–12% sodium dodecyl sulfate polyacrylamide gel electrophoresis followed by transfer to polyvinylidene difluoride membranes and blocking in 5% dry milk reconstituted in PBST (PBS supplemented with Tween-20 at 0.05%), as previously described. Antibodies were diluted in 2.5% dry milk in PBST. For CypD detection, blots were probed with monoclonal CypD antibody (RRID: AB_478283) diluted 1:1 000 and HRP-conjugated goat anti-mouse antibody diluted 1:3 000. To verify equal loading, blots were re-probed with VDAC1 (RRID: AB_632587) antibody diluted 1:2 000 and HRP-conjugated goat anti-mouse antibody diluted 1:5 000. CypD and VDAC1 signals were developed with West Pico Substrate.

### Mitochondrial morphology and functional assays

FSS-induced and control cells, as described above, were stained with the fluorescent nuclear stain, Hoechst 33343 (Molecular Probes H 1339), at 0.5 μmol/L, and the Δψm-dependent dye TMRE (Invitrogen T669) at 20 nmol/L, or MitoTacker Green (Invitrogen M7514) at 100 nmol/L. For TMRE staining, FCCP treatment (Abcam ab120081) at 0.5 μmol/L was used as a negative control. Cells were imaged using an inverted fluorescence microscope (Axioscope), and the fluorescence signal was quantified using ImageJ. For mitochondrial network analysis, MitoTracker Green-stained cells had mitochondria identified in ImageJ using a convolution filter and thresholding for intensity and minimum pixel area as previously described.^44^ Particle analysis detects and calculates measurements for individual mitochondria.

### Maxillary incisor extraction

After IP anesthesia injection—100 mg/kg Ketamine and 10 mg/kg Xylazine—unilateral maxillary incisor extraction was performed on 3-month-old control (*Ppif*^*f/f*^), OB-specific inducible *Ppif* deletion (2.3 kb *Col1*^*CreERt2*^;*Ppif*^*f*/f^), or C57Bl/6J, mice. The surgical procedure was executed with the aid of a stereomicroscope. A 21-gauge needle was used as an elevator to promote the expansion of the dental socket by lateral displacing movement (luxation). The luxation was initiated by repeated smooth movements in the mesiodistal direction and completed by repeated movements in the labiolingual direction. The extraction was achieved using a forceps to pull the tooth occlusally in the lingual direction. Right after extraction, the dental specimen was evaluated on a stereomicroscope to confirm whether the surgical procedure effectively removed the entire dental root without leaving any remnant in the socket. Mice that had not had their tooth entirely removed during the surgical procedure were excluded from this study. Cre recombination was induced after complete socket healing, 28 days post-surgery, and then re-induced 1.5 months later. Both Cre^+^ mice and Cre^−^ control littermates received 100 μL of 3 mg/mL tamoxifen injections intraperitoneally qd × 5. Tissue harvesting was done at 4 months post-surgery.

### Maxillary intra-cortical alveolar AAV2(serotype 2)-Cre-DIO-*caPpif* -eGFP infection

As previously described,^16^ *caPpif* construct was cloned into the Double-floxed Inverted Orientation (DIO) Cre-On vector pAAV-Ef1a-DIO-eGFP-WPRE-pA (Addgene plasmid # 37084; http://n2t.net/addgene:37084; RRID:Addgene_37084—gift from Bernardo Sabatini^39^). DIO vector was cloned using the AscI and NheI restriction sites, and the transgene was introduced through transgene-specific primers and PCR amplification. The AscI site was N-terminal and the NheI site C-terminal with respect to the transgene. Cloning and sequence confirmation were performed. Cloned vectors were amplified with recombination-deficient bacteria (OneShot Stbl3, Invitrogen) and tested functionally for Cre-On expression by calcium phosphate transfection (Invitrogen) into HEK293 cells constitutively expressing Cre. The AAV particles (AAV2-Cre-DIO-*caPpif-*eGFP) were produced at the University of North Carolina Viral Core Facility.

*Col1*^*CreERt2/+*^;*Ppif*^*f/f*^ (AAV-DIO Cre^+^) and Cre-negative *Ppif*^*f/f*^ littermate mice (AAV-DIO Cre^−^) that underwent tooth extraction and went through socket healing for 28 days were anesthetized using 100 mg/kg Ketamine and 10 mg/kg Xylazine IP at a rate of 0.1 mL per 10 grams of body weight. Ten microliters of viral solution (1.8 × 10^13^ viral particles/mL AAV2-Cre-DIO-*caPpif* -eGFP) was slowly injected by free hand intra-cortically in the alveolar region of palatine bone. Tamoxifen (100 μL of 3 mg/mL stock) was intraperitoneally injected qd × 5 in both AAV-DIO Cre^+^ mice and Cre^−^ control littermates.

### Bone micro-computed tomography (microCT)

Following euthanasia, maxillofacial bones were isolated and cleaned of excess soft tissue. Bones were stored at −80 °C prior to μCT. Specimens were imaged using high-resolution acquisition (10.5 μm voxel size) with the VivaCT 40 tomograph (Scanco Medical). Scanco analysis software was utilized for volume quantification.

### Histology

After microCT scanning, bone specimens were NBF-fixed and processed for histology via decalcification in Webb-Jee 14% EDTA solution for 3 weeks, followed by paraffin embedding. Sections were cut to 5 μm in three levels of each sample and then stained with either TRAP or immunohistochemistry (IHC). The cell monolayer was fixed in 4% PFA for 30 min.

### Immunohistochemistry

NBF-fixed bone specimens were processed as above. IHC was carried out using a primary anti-CypD antibody (RRID:AB_10864110) diluted 1:500. The primary antibody solution was composed of PBS, 0.1% Tween-20, and 5% Goat Serum. Prior incubation at 65 °C in 10 mmol/L sodium citrate (pH 6.0) overnight was performed for antigen retrieval. Quenching and biotinylated anti-mouse IgG secondary antibody (RRID:AB_2336528) incubation was done using ImmPRESS® HRP Horse Anti-Mouse IgG Polymer Detection Kit (Peroxidase, MP-7402). Slides were prepared to have both control and extraction side sectioning developed on the same slide. Chromogen detection was visualized with DAB substrate Kit (Abcam, ab64238) and counterstained with Hematoxylin QS. Each DAB signal intensity data point represents one biological replicate (mouse), where three different section levels were counted and averaged.

### Immunofluorescence (IF)

NBF-fixed bone specimens or cell monolayers were processed as described. For bone samples, IF was carried out using a primary anti-cyclophilin F (CypD) antibody (RRID: AB_10864110) diluted 1:400 or an anti-GFP antibody (RRID:AB_303395) diluted 1:500, followed by incubation with an anti-mouse IgG secondary antibody conjugated with Alexa Fluor®647 (RRID:AB_2811129) diluted 1:2 000 and with anti-rabbit IgG secondary antibody conjugated with Alexa Fluor®488 (RRID:AB_2630356), respectively. For cell monolayer samples, CypD IF was performed as above and incubated with the primary anti-runx2 antibody (RRID:AB_2892645) conjugated to Alexa Fluor®647 diluted 1:500, or with anti-ATP5a antibody (RRID:AB_3224415) conjugated to Alexa Fluor®647 diluted 1:400. The primary antibody solution was composed of PBS, 0.1% Tween-20, and 5% Goat Serum. Prior incubation at 65 °C in 10 mmol/L sodium citrate (pH 6.0) for 3 h was performed for antigen retrieval. Fluoroshield Mounting Medium with DAPI (ab104139) was used to counterstain and coverslip IF-slides. In vitro samples: each IF signal intensity data point represents one biological replicate composed of three technical replicates quantified and averaged. In vivo specimens: each IF signal intensity data point represents one biological replicate (mouse), where three different section levels were counted and averaged

### Histomorphometry

TRAP or IF-stained slides were scanned in an Olympus VSL20 whole slide imager at 40× magnification and evaluated with VisioPharm automated histomorphometry software. TRAP-stained slides were analyzed to measure the TRAP-positive area relative to total bone area in the bone specimens. Three different levels were counted per mouse and averaged.

### Statistics

For statistical analysis, Tukey’s multiple comparison test, following ordinary one-way ANOVA, was used for multiple comparisons. For comparisons where the difference of two sample groups was independent, an unpaired *t*-test was used for the frequency distribution of the differences between the two groups that fitted a normal distribution. When the left and right sides (tooth extraction side compared to contralateral-control) from the same animal were compared, we used a paired *t*-test.

## Supplementary information
