# Supplementary material for: Mechano-metabolic axis involves mitochondrial responses via BMP/Smad regulatory effect on cyclophilin D
Source: Bone Res. 2026 Sep 2;14:93. doi: 10.1038/s41413-026-00580-y (PMC13538553; doi:10.1038/s41413-026-00580-y)
Supplement: Supplementary file 1 [file 41413_2026_580_MOESM1_ESM.pdf]

Fig.S1

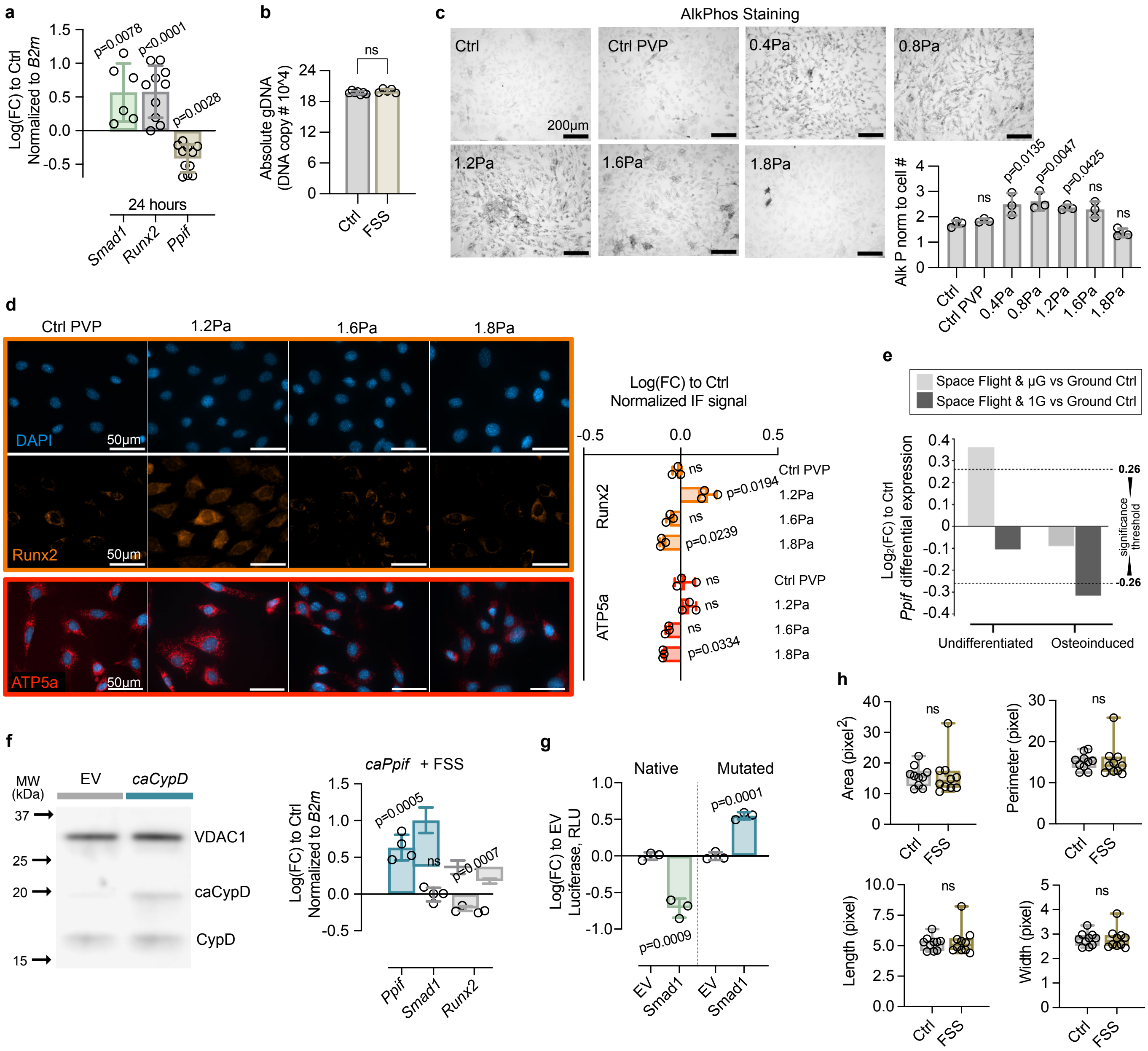
a
Log(FC) to Ctrl
Normalized to B2m
p=0.0078
p<0.0001
p=0.0028
24 hours
Smad1
Runx2
Ppif
b
Absolute gDNA
(DNA copy # 10^4)
ns
Ctrl
FSS
c
AlkPhos Staining
Ctrl
Ctrl PVP
0.4Pa
0.8Pa
1.2Pa
1.6Pa
1.8Pa
200µm
Alk P norm to cell #
p=0.0135
p=0.0047
p=0.0425
ns
d
Ctrl PVP
1.2Pa
1.6Pa
1.8Pa
DAPI
Runx2
ATP5a
50µm
Log(FC) to Ctrl
Normalized IF signal
p=0.0194
p=0.0239
p=0.0334
e
Space Flight & µG vs Ground Ctrl
Space Flight & 1G vs Ground Ctrl
Log2(FC) to Ctrl
Ppif differential expression
significance threshold
0.26
-0.26
Undifferentiated
Osteoinduced
f
MW
(kDa)
EV
caCypD
37
25
20
15
VDAC1
caCypD
CypD
caPpif + FSS
Log(FC) to Ctrl
Normalized to B2m
p=0.0005
p=0.0007
Ppif
Smad1
Runx2
g
Native
Mutated
Log(FC) to EV
Luciferase, RLU
p=0.0001
p=0.0009
EV
Smad1
h
Area (pixel²)
Perimeter (pixel)
Length (pixel)
Width (pixel)
