# Supplementary material for: Mechano-metabolic axis involves mitochondrial responses via BMP/Smad regulatory effect on cyclophilin D
Source: Bone Res. 2026 Sep 2;14:93. doi: 10.1038/s41413-026-00580-y (PMC13538553; doi:10.1038/s41413-026-00580-y)
Supplement: Supplementary file 2 [file 41413_2026_580_MOESM2_ESM.pdf]

Fig.S2

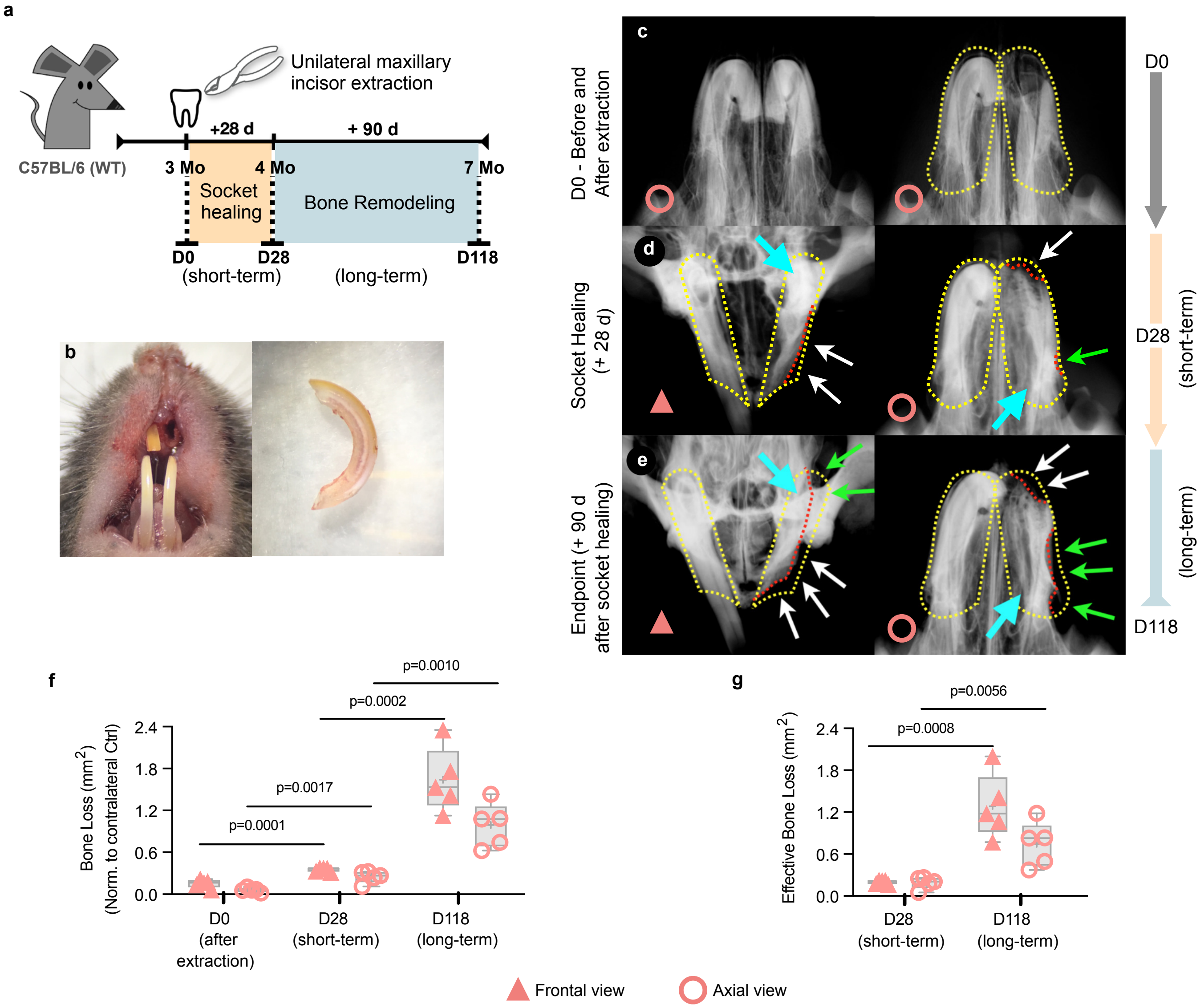

a
C57BL/6 (WT)
Unilateral maxillary incisor extraction
+28 d
+ 90 d
3 Mo
4 Mo
7 Mo
Socket healing
Bone Remodeling
D0
D28
D118
(short-term)
(long-term)
b
c
d
e
D0 - Before and After extraction
Socket Healing (+ 28 d)
Endpoint (+ 90 d after socket healing)
D0
D28
(short-term)
(long-term)
D118
f
Bone Loss (mm²) (Norm. to contralateral Ctrl)
2.4
1.8
1.2
0.6
0.0
p=0.0010
p=0.0002
p=0.0017
p=0.0001
D0 (after extraction)
D28 (short-term)
D118 (long-term)
g
Effective Bone Loss (mm²)
p=0.0056
p=0.0008
D28 (short-term)
D118 (long-term)
Frontal view
Axial view
