# Supplementary material for: Mechano-metabolic axis involves mitochondrial responses via BMP/Smad regulatory effect on cyclophilin D
Source: Bone Res. 2026 Sep 2;14:93. doi: 10.1038/s41413-026-00580-y (PMC13538553; doi:10.1038/s41413-026-00580-y)
Supplement: Supplementary file 3 [file 41413_2026_580_MOESM3_ESM.pdf]

Fig.S3

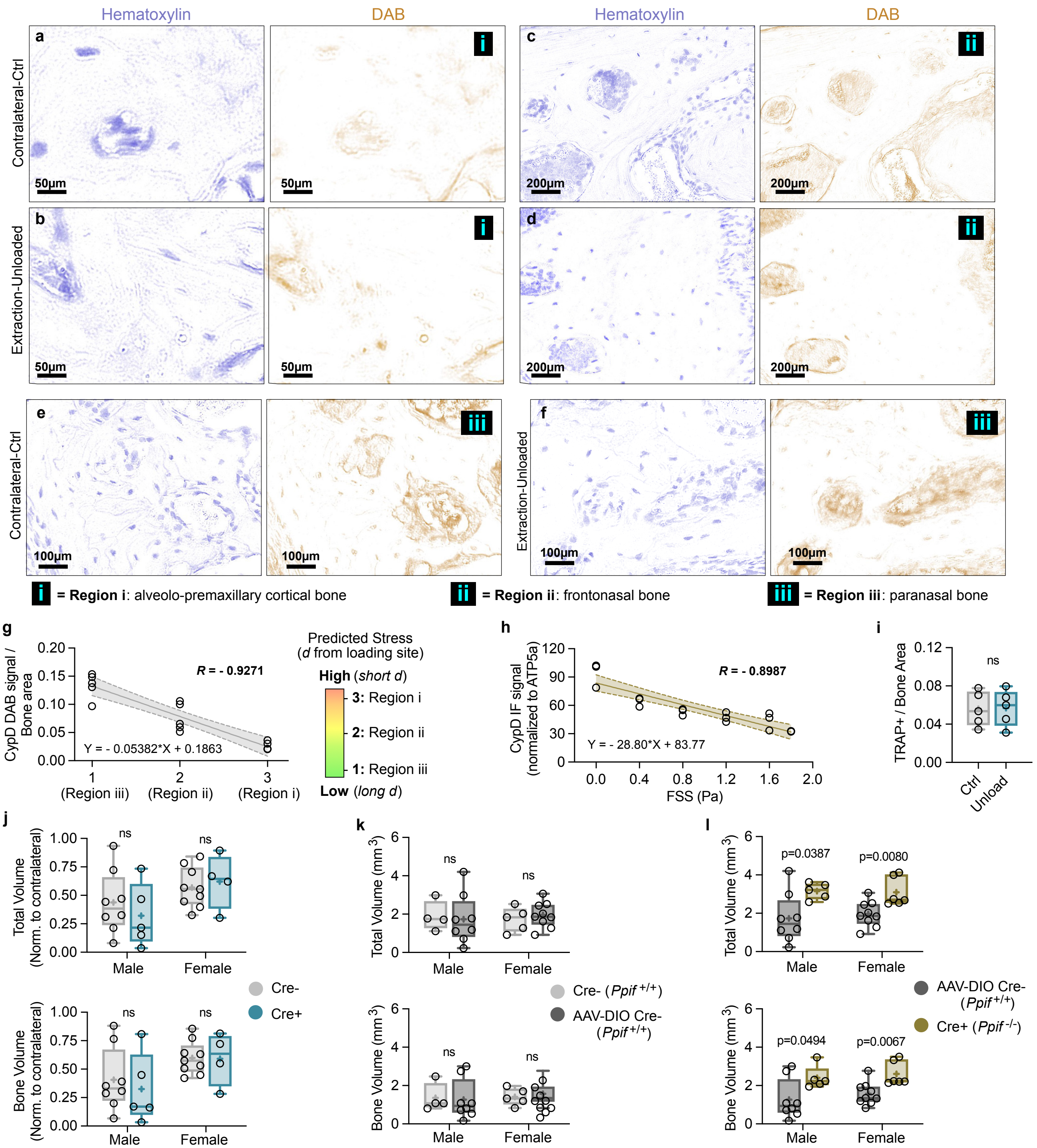

Hematoxylin
DAB
Hematoxylin
DAB
a
b
c
d
e
f
i
ii
iii
Contralateral-Ctrl
Extraction-Unloaded
50µm
200µm
100µm
i = Region i: alveolo-premaxillary cortical bone
ii = Region ii: frontonasal bone
iii = Region iii: paranasal bone
g
CypD DAB signal / Bone area
R = - 0.9271
Y = - 0.05382*X + 0.1863
1 (Region iii)
2 (Region ii)
3 (Region i)
Predicted Stress
(d from loading site)
High (short d)
3: Region i
2: Region ii
1: Region iii
Low (long d)
h
CypD IF signal
(normalized to ATP5a)
R = - 0.8987
Y = - 28.80*X + 83.77
FSS (Pa)
i
TRAP+ / Bone Area
ns
Ctrl
Unload
j
Total Volume
(Norm. to contralateral)
Bone Volume
(Norm. to contralateral)
Male
Female
Cre-
Cre+
k
Total Volume (mm3)
Bone Volume (mm3)
Cre- (Ppif +/+)
AAV-DIO Cre- (Ppif +/+)
l
p=0.0387
p=0.0080
p=0.0494
p=0.0067
AAV-DIO Cre- (Ppif +/+)
Cre+ (Ppif -/-)
