# Supplementary material for: Mechano-metabolic axis involves mitochondrial responses via BMP/Smad regulatory effect on cyclophilin D
Source: Bone Res. 2026 Sep 2;14:93. doi: 10.1038/s41413-026-00580-y (PMC13538553; doi:10.1038/s41413-026-00580-y)
Supplement: Supplementary file 4 [file 41413_2026_580_MOESM4_ESM.pdf]

Fig.S4

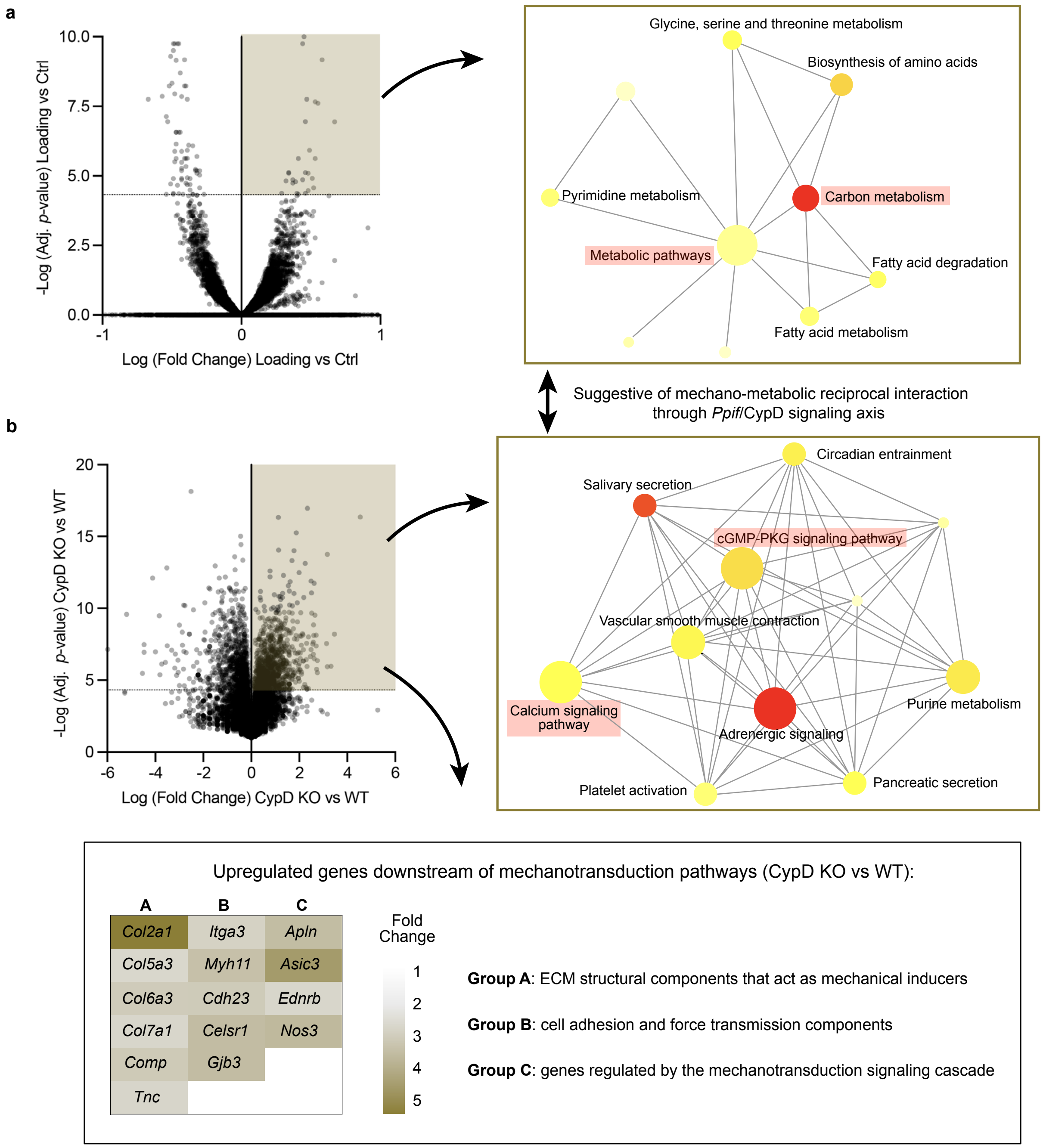

a
10.0
7.5
5.0
2.5
0.0
-1
0
1
-Log (Adj. p-value) Loading vs Ctrl
Log (Fold Change) Loading vs Ctrl
Glycine, serine and threonine metabolism
Biosynthesis of amino acids
Pyrimidine metabolism
Carbon metabolism
Metabolic pathways
Fatty acid degradation
Fatty acid metabolism
Suggestive of mechano-metabolic reciprocal interaction
through Ppif/CypD signaling axis
b
20
15
10
5
0
-6
-4
-2
0
2
4
6
-Log (Adj. p-value) CypD KO vs WT
Log (Fold Change) CypD KO vs WT
Circadian entrainment
Salivary secretion
cGMP-PKG signaling pathway
Vascular smooth muscle contraction
Calcium signaling pathway
Adrenergic signaling
Purine metabolism
Platelet activation
Pancreatic secretion
Upregulated genes downstream of mechanotransduction pathways (CypD KO vs WT):
A
B
C
Col2a1
Itga3
Apln
Col5a3
Myh11
Asic3
Col6a3
Cdh23
Ednrb
Col7a1
Celsr1
Nos3
Comp
Gjb3
Tnc
Fold Change
1
2
3
4
5
Group A: ECM structural components that act as mechanical inducers
Group B: cell adhesion and force transmission components
Group C: genes regulated by the mechanotransduction signaling cascade
