# Supplementary material for: Mechano-metabolic axis involves mitochondrial responses via BMP/Smad regulatory effect on cyclophilin D
Source: Bone Res. 2026 Sep 2;14:93. doi: 10.1038/s41413-026-00580-y (PMC13538553; doi:10.1038/s41413-026-00580-y)
Supplement: Supplementary file 5 [file 41413_2026_580_MOESM5_ESM.pdf]

Fig.S5

2.3Kb *Col1*^{CreERt2};*Ppif*$^{f/f}$

		Total Vol. (mm3)	Bone Vol. (mm3)	BV/TV	Tb.Th (mm)	Tb.N (1/mm)	Tb.Sp (mm)	Tb BMD (mgHA/ccm)	Ct.Th (mm)	Ct BMD (mgHA/ccm)
**MALE**	Cre- M_01	1.1135	0.8071	0.7248	0.1034	9.1810	0.0789	916.0395	0.1310	1125.8868
	Cre- M_02	1.7167	1.0836	0.6312	0.0960	8.2610	0.1049	878.6784	0.1370	1100.1556
	Cre- M_03	1.7702	1.0694	0.6041	0.0984	8.3060	0.1224	849.2402	0.1170	1069.3363
	Cre- M_04	2.9824	2.4775	0.8307	0.0998	7.6492	0.1091	1133.8824	0.1560	1133.8824
	**Average**	1.8957	1.3594	0.6977	0.0994	8.3493	0.1038	944.4601	0.1353	1107.3153
	**Std Dv**	0.7833	0.7562	0.1026	0.0031	0.6302	0.0182	129.2060	0.0162	29.1226
	Cre+ M_01	3.2226	2.2326	0.6928	0.1303	7.0700	0.1411	932.8301	0.1090	1109.3868
	Cre+ M_02	3.4707	2.3078	0.6649	0.0908	9.8326	0.0836	895.6871	0.1050	1097.9023
	Cre+ M_03	3.6211	3.4728	0.9590	0.1099	9.1632	0.0940	929.4139	0.1360	1028.5590
	Cre+ M_04	2.5923	2.0855	0.8045	0.1046	10.3660	0.0682	952.9645	0.1210	1121.9618
	Cre+ M_05	2.8758	1.9461	0.7803	0.0974	9.2733	0.1003	879.4780	0.1040	1086.6359
	**Average**	3.1565	2.4090	0.7803	0.1066	9.1410	0.0974	918.0747	0.1150	1088.8892
	**Std Dv**	0.4231	0.6107	0.1157	0.0151	1.2536	0.0273	29.8098	0.0135	36.1939
	***P*-value**	0.0171	0.0541	0.3009	0.3851	0.2910	0.7012	0.6668	0.0796	0.4373
**FEMALE**	Cre- F_01	1.8338	1.3239	0.7219	0.1165	8.7839	0.1134	916.1121	0.1120	1118.3274
	Cre- F_02	2.0306	1.7065	0.8404	0.1454	8.9963	0.0757	953.0372	0.1130	1124.9419
	Cre- F_03	1.2792	1.1999	0.9380	0.2099	7.5682	0.0703	1005.2991	0.1160	1076.3143
	Cre- F_04	0.9164	0.8293	0.9050	0.1571	9.3566	0.0659	924.8347	0.1350	1052.4003
	Cre- F_05	2.5288	1.9750	0.8513	0.1264	9.0818	0.1056	867.6299	0.1160	1016.4930
	**Average**	1.7178	1.4069	0.8513	0.1511	8.7574	0.0862	933.3826	0.1184	1077.6954
	**Std Dv**	0.6332	0.4461	0.0826	0.0365	0.6957	0.0217	50.6286	0.0094	45.4716
	Cre+ F_01	2.7448	2.2050	0.8033	0.1616	7.4704	0.1130	974.5526	0.1300	1141.1511
	Cre+ F_02	2.5472	2.2324	0.8764	0.1476	9.0388	0.0815	929.4866	0.1250	1099.7195
	Cre+ F_03	4.1197	3.5073	0.8513	0.1545	9.0113	0.0895	899.1035	0.1030	1092.1600
	Cre+ F_04	4.0041	3.3272	0.8310	0.2409	6.1827	0.0924	987.2728	0.1230	1171.3163
	Cre+ F_05	2.5108	2.2162	0.8405	0.2131	7.3668	0.0923	996.9401	0.1330	1125.0873
	Cre+ F_06	2.6792	2.1367	0.7975	0.1665	8.1860	0.1285	853.1652	0.1100	1043.0237
	**Average**	3.1010	2.6041	0.8333	0.1807	7.8760	0.0995	940.0868	0.1207	1112.0763
	**Std Dv**	0.7501	0.6332	0.0297	0.0375	1.0980	0.0176	56.5581	0.0117	44.3658
	***P*-value**	0.0098	0.0063	0.6286	0.2191	0.1562	0.2886	0.8421	0.7365	0.2374
