# Supplementary material for: Mechano-metabolic axis involves mitochondrial responses via BMP/Smad regulatory effect on cyclophilin D
Source: Bone Res. 2026 Sep 2;14:93. doi: 10.1038/s41413-026-00580-y (PMC13538553; doi:10.1038/s41413-026-00580-y)
Supplement: Supplementary file 6 [file 41413_2026_580_MOESM6_ESM.pdf]

Fig.S6

2.3Kb *Col1*^{CreERt2};*Ppif*$^{f/f}$ + AAV-DIO

		Total Vol. (mm3)	Bone Vol. (mm3)	BV/TV	Tb.Th (mm)	Tb.N (1/mm)	Tb.Sp (mm)	Tb BMD (mgHA/ccm)	Ct.Th (mm)	Ct BMD (mgHA/ccm)
**MALE**	Cre- M_01	0.7128	0.5374	0.7540	0.1032	9.1547	0.0865	907.8985	0.1390	1090.0521
	Cre- M_02	4.2012	3.0096	0.7164	0.0923	7.4376	0.0713	1083.1469	0.1340	1083.1469
	Cre- M_03	0.2241	0.1652	0.7371	0.0809	11.4613	0.0663	887.8370	0.1240	1081.3297
	Cre- M_04	1.1797	0.7180	0.6086	0.0896	7.8074	0.1383	886.1651	0.1530	1112.0037
	Cre- M_05	1.1135	0.8071	0.7248	0.1034	9.1810	0.0789	916.0395	0.1310	1125.8868
	Cre- M_06	1.7167	1.0836	0.6312	0.0960	8.2610	0.1049	878.6784	0.1370	1100.1556
	Cre- M_07	1.7702	1.0694	0.6041	0.0984	8.3060	0.1224	849.2402	0.1170	1069.3363
	Cre- M_08	2.9824	2.4775	0.8307	0.0998	7.6492	0.1091	1133.8824	0.1560	1133.8824
	**Average**	1.7376	1.2335	0.7009	0.0955	8.6573	0.0972	942.8610	0.1364	1099.4742
	**Std Dv**	1.2907	0.9874	0.0798	0.0076	1.3033	0.0256	105.0424	0.0133	22.7780
	Cre+ M_01	0.6027	0.3634	0.6030	0.0791	9.9060	0.0882	869.8832	0.1070	1008.3520
	Cre+ M_02	1.8650	1.4903	0.7991	0.0815	7.8921	0.1123	1071.2990	0.1070	1071.2990
	Cre+ M_03	0.4658	0.3866	0.8300	0.1403	8.8217	0.0799	943.3698	0.1510	1099.1381
	Cre+ M_04	0.0920	0.0601	0.6537	0.0621	12.8095	0.0762	893.4330	0.1560	987.3454
	Cre+ M_05	3.2957	2.9669	0.9002	0.1161	8.3122	0.0892	1104.0807	0.1410	1104.0807
	**Average**	1.2642	1.0535	0.7572	0.0958	9.5483	0.0892	976.4131	0.1324	1054.0430
	**Std Dv**	1.3169	1.2000	0.1245	0.0317	1.9725	0.0141	105.6273	0.0238	53.3183
	***P*-value**	0.5362	0.7733	0.3369	0.9747	0.3438	0.5365	0.5873	0.7033	0.0539
**FEMALE**	Cre- F_01	3.0659	2.7758	0.9054		8.4395		1128.9397	0.1490	1128.9397
	Cre- F_02	2.4449	1.9115	0.7818	0.1600	7.6184	0.1113	949.7663	0.1650	1077.1139
	Cre- F_03	1.8650	1.3519	0.7249	0.1303	7.0952	0.1299	956.1628	0.1670	1125.0873
	Cre- F_04	1.6224	1.1007	0.6784	0.1005	9.6225	0.0959	849.6036	0.1270	1051.8187
	Cre- F_05	1.8338	1.3239	0.7220	0.1165	8.7839	0.1134	916.1121	0.1120	1118.3274
	Cre- F_06	2.0306	1.7065	0.8404	0.1454	8.9963	0.0757	953.0372	0.1130	1124.9419
	Cre- F_07	1.2792	1.1999	0.9380	0.2099	7.5682	0.0703	1005.2991	0.1160	1076.3143
	Cre- F_08	0.9164	0.8293	0.9049	0.1571	9.3566	0.0659	924.8347	0.1350	1052.4003
	Cre- F_09	2.5288	1.9750	0.7810	0.1264	9.0818	0.1056	867.6299	0.1160	1016.4930
	**Average**	1.9541	1.5749	0.8085	0.1433	8.5069	0.0960	950.1539	0.1333	1085.7152
	**Std Dv**	0.6593	0.5881	0.0931	0.0336	0.8861	0.0232	81.9835	0.0221	40.6721
	Cre+ F_01	1.1519	0.9559	0.8298	0.1331	9.1752	0.0902	909.4250	0.1630	1095.8671
	Cre+ F_02	3.9374	3.2688	0.8302	0.1243	8.7573	0.1114	1121.0895	0.1680	1121.0895
	Cre+ F_03	2.6837	2.5385	0.9459	0.1140	8.8708	0.0911	1075.2240	0.1440	1075.2240
	Cre+ F_04	2.7047	2.1139	0.7816	0.1492	7.7450	0.1437	1004.7176	0.1240	1004.7176
	**Average**	2.6194	2.2193	0.8469	0.1301	8.6371	0.1091	1027.6140	0.1498	1074.2246
	**Std Dv**	1.1405	0.9679	0.0698	0.0149	0.6203	0.0251	92.1905	0.0200	49.9898
	***P*-value**	0.2036	0.1603	0.4805	0.4814	0.7971	0.3889	0.1571	0.2307	0.6681
