# Supplementary material for: Mechano-metabolic axis involves mitochondrial responses via BMP/Smad regulatory effect on cyclophilin D
Source: Bone Res. 2026 Sep 2;14:93. doi: 10.1038/s41413-026-00580-y (PMC13538553; doi:10.1038/s41413-026-00580-y)
Supplement: Supplementary file 7 [file 41413_2026_580_MOESM7_ESM.docx]

**FIGURE SUPPLEMENT LEGENDS**

**Fig. S1. Mechanical loading downstream effects are dependent on *Ppif* mRNA expression and CypD protein levels.** MC3T3-e1 calvaria-derived cells were subjected to 1.2 Pa of FSS intermittently at 0.2 Hz for 1 hour. Non-induced cells were used as controls. **a**) Real-time RT-qPCR data on RNA collected 24 hours after 1.2 Pa FSS induction: *Smad1* and *Runx2,* osteogenic markers, were upregulated, whereas *Ppif* was downregulated; **b**) Analysis of DNA copy number showed no differences between FSS and control cells, indicating no substantial proliferation; **c**) Alkaline phosphatase staining showed higher intensity for induced cells under 0.4 to 1.2Pa of FSS when compared to controls. Non-induced control with or without polyvinylpyrrolidone (PVP) presented a similar AlkPhos signal; **d**) IF-stained panel of representative images comparing the protein levels of Runx2, ATP5a, and CypD at 1,2, 1.6 and 1.8Pa FSS levels vs. their respective control. No changes in Runx2 and ATP5a were found for the 1.6Pa condition, while 1.8Pa of FSS not only prevented Runx2 stabilization but it also decreased Runx2 and ATP5a protein levels; **e**) Publicly available transcriptomic data from NASA (https://genelab-data.ndc.nasa.gov/genelab/accession/GLDS-29/) were analyzed: both undifferentiated or osteoinduced BMSCs showed higher *Ppif* expression in microgravity conditions; **f**) MC3T3-e1 cells were transfected with pCMV6-*caPpif* vector to express constitutively active CypD K166Q mutant and thus achieve CypD gain-of-function (GOF) prior to FSS induction. Representative western blot of stably transfected cells (EV: pCMV6-empty vector; caCypD: pCMV6-*caPpif* vector; VDAC1: loading control; caCypD: mutant CypD; CypD: endogenous CypD). RT-qPCR: CypD GOF shows higher *Ppif* expression and disrupted osteogenic cascade activation when compared to pCMV6 empty vector-transfected cells; **g**) Dual luciferase reporter assay demonstrating the specificity of the 1.1kb *Ppif*-Luc reporter: Smad1 co-transfection of MC3T3-e1 cells with mouse native-derived 1.1kb *Ppif* promoter downregulated luciferase signal. Contrary, Smad1 co-transfection with 1.1 kb SBE-mutated *Ppif-*luc reporter rescued luciferase signal; **h**) Mitochondria stained with MitoTracker Green were identified in ImageJ using a convolution filter and thresholding for intensity and minimum pixel area. Particle analysis detected and measured individual mitochondrion. No differences were found in the following parameters: Mitochondrial area, mitochondrial perimeter, mitochondrial length, and mitochondrial width. Plots display the actual data points from independent biological replicates; Bar graphs report mean ± SD; *P* values determined by Tukey’s multiple comparison test following ordinary one-way ANOVA in multiple comparisons, or an unpaired *t*-test for a single continuous variable comparison.

**Fig. S2. Tooth extraction-mediated unloading leads to alveolar and maxillary bone loss in a murine model.** Unilateral maxillary incisor extraction was performed in 3Mo-old C57BL/6 mice and followed up for up to 4Mo. **a**) Experimental design: short-term time point was set as 28 days post-surgery and designated as socket healing stage as previously reported^(34)^. Long-term time point was set as 90 days post-socket healing (118 days/4Mo post-surgery) depicting the stage of interest for bone remodeling changes in our model; **b**) Occlusal view after dental extraction (right). Extracted incisor showing no fractures or cracks (left); **c**) Axial x-ray before dental extraction (top left), and right after dental extraction (top right); **d**) Posterior-Anterior (frontal) and axial x-ray after completed socket healing, 28 days post-surgery; **e**) Posterior-Anterior (frontal) and axial x-ray 118 days (4Mo) post-surgery. Yellow line represents the actual or expected bone area when compared to the contralateral control side. Red line depicts the bone resorption surface. White arrows point to the alveolar bone. Green arrows point to the maxillary basal bone. Blue arrow shows there is no dental remnant in the apical area that could cause incisor regrowth; **f**) Bone loss quantification on the extraction side normalized to contralateral control, demonstrating that under mechanical unloading conditions, there is a steady process of bone loss; **g**) Effective bone loss is calculated by subtracting the loss observed right after extraction that is due to the technical procedure itself. The long-term bone loss is substantially higher than that observed at the short-term. Boxplots display the actual data points from five independent mice, showing median, mean, and range; *P* values determined by Tukey’s multiple comparison test following ordinary one-way ANOVA.

**Fig. S3. CypD expression is inversely correlated with the magnitude of mechanical stress.** **a-f**) Image color deconvolution from IHC images shown in Figure 3 depicting hematoxylin and DAB staining separately. **a-f**) panels are sequentially arranged from 3a.i, 3b.i, 3d.ii, 3e.ii, 3d.iii, and 3d.iii; **g**) Linear regression of CypD expression *in vivo* data over predicted stress intensity (or *d* distance from incisor loading site) showing a strong inverse linear relationship; **h**) Linear regression of CypD expression in vitro data over FSS intensity demonstrating a strong inverse linear relationship; **i**) No changes in TRAP+ staining per bone area were observed when Contralateral-Ctrl was compared to Extraction-Unloaded side; **j**) Total alveolar volume, alveolar bone volume normalized to Contralateral-Ctrl presented no differences in control AAV-DIO Cre^-^ mice compared to Cre^+^ CypD GOF; **k**) Cre^-^ (*Ppif* ^+/+^) and AAV-DIO Cre^-^ (*Ppif* ^+/+^) showed no statistical differences in total alveolar and bone volume; **l**) *Ppif* deletion invariably showed protection against total alveolar and bone volume even when compared to AAV-DIO Cre^-^ (*Ppif* ^+/+^). Boxplots display the actual data points from independent biological replicates, showing median, mean, and range; *P* values determined by Tukey’s multiple comparison test following ordinary one-way ANOVA in multiple comparisons, or an unpaired *t*-test for a single continuous variable comparison.

**Fig. S4. RNAseq data analysis revealed a suggestive reciprocal interaction between mechano-stimulated cells and *Ppif* deletion. a**) Publicly available transcriptomic data comparing mechano-stimulated vs. non-stimulated BMSCs were analyzed (https://doi.org/10.1016/j.bbrep.2024.101847). Data from cells subjected to mechanical loading assayed 24h after stimuli vs. controls are shown in the volcano plot. The upregulated differentially expressed genes list was input into a pathway analysis, which revealed metabolic pathways and carbon metabolism among the top pathway hits for the early stimulus response; **b**) Transcriptomic data comparing *Ppif* KO BMSCs vs. control were analyzed (https://doi.org/10.1016/j.bone.2020.115391). The upregulated differentially expressed genes list pathway analysis showed *Ppif* KO BMSCs present enrichment gene signature similar to that in cells with activated mechanotransduction pathways.

**Fig. S5. Alveolar bone volumetric parameters in mice with osteoblast-specific *Ppif* conditional deletion preceded by unilateral maxillary incisor extraction.** Bone volumetric and biomechanical properties of 2.3kb *Col1^CreERt2^;Ppif^f/f^* mice were measured by microCT. No differences in the following bone morphological parameters were found between groups: BV/TV (bone fractional volume), Tb.Th (trabecular thickness), Tb.N (trabecular number), Tb.Sp (trabecular space), Tb. BMD (trabecular bone mineral density), Ct.Th (cortical thickness), Ct. BMD (cortical bone mineral density). Calculated means and *P* value determined by an unpaired *t*-test.

**Fig. S6. Alveolar bone volumetric parameters in mice with osteoblast-specific *Ppif* conditional deletion with *caPpif* re-expression preceded by unilateral maxillary incisor extraction.** Bone volumetric and biomechanical properties of 2.3kb *Col1^CreERt2^;Ppif^f/f^* mice with *caPpif* re-expression (AAV-DIO ca*Ppif*) were measured by microCT. No differences in the following bone morphological parameters were found between groups in BV/TV (bone fractional volume), Tb.Th (trabecular thickness), Tb.N (trabecular number), Tb.Sp (trabecular space), Tb. BMD (trabecular bone mineral density), Ct.Th (cortical thickness), and Ct. BMD (cortical bone mineral density). Calculated means and *P* value determined by an unpaired *t*-test.
